# A Nomogram Based on a Three-Gene Signature Derived from AATF Coexpressed Genes Predicts Overall Survival of Hepatocellular Carcinoma Patients

**DOI:** 10.1155/2020/7310768

**Published:** 2020-04-22

**Authors:** Jun Liu, Jianjun Lu, Zhanzhong Ma, Wenli Li

**Affiliations:** ^1^Departments of Clinical Laboratory, Yue Bei People's Hospital, Shantou University Medical College, Shaoguan, Guangdong, China; ^2^The Second School of Clinical Medicine, Southern Medical University, Guangzhou, Guangdong, China; ^3^Department of Medical Services, First Affiliated Hospital of Sun Yat-sen University, Guangzhou, Guangdong, China; ^4^Departments of Reproductive Medicine Center, Yue Bei People's Hospital, Shantou University Medical College, Shaoguan, Guangdong, China

## Abstract

**Background:**

Hepatocellular carcinoma (HCC) is a common cancer with an extremely high mortality rate. Therefore, there is an urgent need in screening key biomarkers of HCC to predict the prognosis and develop more individual treatments. Recently, AATF is reported to be an important factor contributing to HCC.

**Methods:**

We aimed to establish a gene signature to predict overall survival of HCC patients. Firstly, we examined the expression level of AATF in the Gene Expression Omnibus (GEO), the Cancer Genome Atlas (TCGA), and the International Union of Cancer Genome (ICGC) databases. Genes coexpressed with AATF were identified in the TCGA dataset by the Poisson correlation coefficient and used to establish a gene signature for survival prediction. The prognostic significance of this gene signature was then validated in the ICGC dataset and used to build a combined prognostic model for clinical practice.

**Results:**

Gene expression data and clinical information of 2521 HCC patients were downloaded from three public databases. AATF expression in HCC tissue was higher than that in matched normal liver tissues. 644 genes coexpressed with AATF were identified by the Poisson correlation coefficient and used to establish a three-gene signature (KIF20A, UCK2, and SLC41A3) by the univariate and multivariate least absolute shrinkage and selection operator Cox regression analyses. This three-gene signature was then used to build a combined nomogram for clinical practice.

**Conclusion:**

This integrated nomogram based on the three-gene signature can predict overall survival for HCC patients well. The three-gene signature may be a potential therapeutic target in HCC.

## 1. Introduction

Hepatocellular carcinoma (HCC) is a common cancer with an extremely high mortality rate all over the world [1]. The effect of drug treatment is very limited, and surgical treatment is the main method for HCC. However, the recurrence rate after surgery is usually high, leading to a poor prognosis of HCC patients [2]. In order to improve the clinical outcomes, an effective method to evaluate the prognosis is urgently needed. Heterogeneity in the incidence and clinical manifestations of HCC make it difficult to predict the survival possibility. Moreover, a biomarker with prognostic significance can provide potential therapeutic targets of HCC [3].

The apoptotic antagonistic transcription factor (AATF), also known as che-1, is a highly conserved transcription factor in eukaryotes. It is a major mediator of cellular responses that promotes cell proliferation by inducing cell cycle arrest, autophagy, DNA repair, and apoptosis inhibition. AATF is proved to be an important factor contributing to HCC in the formation and progression in previous studies [4]. AATF interacts with signal transducer and activator of transcription 3 to increase the expression of monocyte chemoattractant protein-1, thereby participating in processes including cell proliferation, migration, invasion colony formation, and anchorage-dependent growth.

In the past decades, many biomarkers such as epithelial cell adhesion molecules [5], CD24 [6], and TGF-*β* [7] have been found to be associated with the prognosis of HCC. Subsequently, multigene signatures constructed by several prognostic-related genes have been verified to be effective biomarkers with prognostic value [8]. AATF plays an important role in the occurrence and development of HCC. Therefore, we aimed to identify a prognostic gene signature based on AATF in HCC by bioinformatics methods and cell experiments. Firstly, we examined the AATF expression with mRNA expression data from seven series in the Gene Expression Omnibus (GEO), the Cancer Genome Atlas (TCGA), and the International Union of Cancer Genome (ICGC) databases. Then, we verified the prognostic value of AATF in HCC and identified coexpressed genes with AATF by the Poisson coefficient in the TCGA dataset. The range of coexpressed genes with AATF was narrowed by LOSSO regression to establish a prognostic gene signature. This gene signature was validated in an independent external HCC dataset from the ICGC. Moreover, the identified genes were validated in an HCC cell line HepG2. Finally, we built a nomogram for clinical practice by combining various prognostic-related clinicopathological factors. In summary, we aim to build a novel predictive tool of survival prediction for individual patient with HCC by bioinformatics methods in the present study.

## 2. Materials and Methods

### 2.1. Data Source

Gene expression data of nine GEO series (GSE22058, January 18, 2013; GSE36376, August 13, 2018; GSE14520, August 20, 2019; GSE54236, March 28, 2019; GSE63898, March 21, 2019; GSE64041, July 26, 2018; and GSE76427, August 13, 2018) were downloaded from the GEO database. A training dataset used to construct a prognosis gene signature was downloaded from the TCGA database including mRNA expression data and clinical information of HCC patients. Meanwhile, a validation dataset used to verify the gene signature was downloaded from the ICGC also including mRNA expression profiles and clinical information of HCC patients. The above databases are publicly available and open access. And the present study followed the data access policies and publishing guidelines of these databases. Therefore, local ethics committees were not required to approve this study.

### 2.2. Exploring the Prognostic Significance of AATF by Kaplan-Meier Curves

The expression level of AATF in HCC tissues was compared with that in adjacent nontumor tissues (ANTT). And the prognostic significance of AATF in HCC was evaluated by Kaplan-Meier curves using datasets from the TCGA and ICGC. Subsequently, the relationship between the AATF expression and clinicopathological features and prognosis of HCC was explored by univariate and multivariate Cox regression analyses.

### 2.3. Coexpressed Genes with AATF

Coexpressed genes with AATF were identified by the Poisson coefficient (coefficient > 0.6, *P* < 0.001) using the TCGA dataset.

### 2.4. Enrichment Analysis

Gene Ontology (GO) and Kyoto Encyclopedia of Genes and Genomes (KEGG) were conducted to coexpressed genes with AATF.

### 2.5. Constructing a Three-Gene Signature for Predicting Overall Survival of HCC Patients

We used univariate, least absolute shrinkage and selection operator (LASSO), and multivariate Cox regression analyses to identify a gene signature from AATF coexpressed genes for predicting overall survival of HCC patients. Specifically, genes with prognostic value were identified by univariate Cox regression analysis and then narrowed down by LASSO Cox regression.

A risk score based on the gene signature was calculated by multiplying the regression coefficient of the multivariate Cox regression model (*β*) with its expression level. The prognostic index (Pi) = (expression level of *β*∗KIF20A) + (expression level of *β*∗UCK2) + (expression level of *β*∗SLC41A3). Using the risk scores, 365 patients from the TCGA dataset were divided into low-risk and high-risk groups. Kaplan-Meier curves were used to compare the prognosis of the two groups above. Meanwhile, the receiver operating characteristic (ROC) curves were used to evaluate the specificity and sensitivity of the gene signature.

Moreover, the gene expression profiling interactive analysis (GEPIA) database (http://gepia.Cancer-pku.cn) [9] was used to verify the prognostic value of KIF20A, UCK2, and SLC41A3. The log-rank test was used to compare the overall survival between the high- and low-mRNA expression groups. *P* < 0.05 indicates a statistically significant difference.

In addition, HCC inpatients of the TCGA dataset were divided into high- and low-risk groups by the risk score. Gene Set Enrichment Analysis (GSEA) was conducted to identify the pathways in which genes of the high-risk group enriched (GSEA software: http://www.broadinstitute.org/gsea/index.jsp).

### 2.6. Verifying the Expression of KIF20A, UCK2, and SLC41A3 by Real-Time Quantitative Polymerase Chain Reaction (RT-qPCR)

The human HCC cell line HepG2 and the normal human liver cell line LO2 were provided by our laboratory and stored in a -80°C refrigerator. In cell experiments, we verified the expression of KIF20A, UCK2, and SLC41A3 by RT-qPCR. Total RNA was extracted from the cells with 2.0 ml TRIzol (Wanleibio Co. Ltd., Shanghai, China) and synthesized into cDNA using the reverse transcriptase M-MLV kit (Wanleibio Co. Ltd., Shanghai, China). The expression levels of KIF20A, UCK2, and SLC41A3 were detected by qPCR using the SYBR Green I kit (Solarbio Co. Ltd., Beijing, China). In this experiment, real time-quantitative polymerase chain reaction (RT-qPCR) was performed using a CFX96 fluorescence quantitative meter (Bio-Rad Inc., California, United States).

*β*-Actin: forward primer 5′-GGCACCCAGCACAATGAA-3′ and reverse primer 5′-CGGACTCGTCATACTCCTGCT-3′, SLC41A: forward primer 5′-CACAAAGATAGTCGGTATCTGACG-3′ and reverse primer 5′-GACCATGGCCAGGATGATT-3′, KIF20A: forward primer 5′-CAGACTTTGCGGCTATGCGA and reverse primer 5′-CTGGTTCTTACGACCCACTTTTA, and UCK2: forward primer 5′-GGACTATCGCCAGAAGCAGGT and reverse primer 5′-CGGACTCGTCATACTCCTGCT. *β*-Actin mRNA was used as an internal control.

The cycling conditions were an initial PCR activation step for 3 minutes at 95°C followed by 40 cycles of three-step amplification at 95°C for 10 seconds, 60°C for 20 seconds, and 72°C for 30 seconds of single acquisition for denaturation and extension. Finally, melting from 65°C to 95°C, every 0.5°C, 5 s. The relative quantifications of KIF20A, UCK2, and SLC41A3 were calculated by the 2^-*ΔΔ*CT^, and each sample was detected for at least 3 times.

### 2.7. Validation of the Three-Gene Signature in the ICGC Dataset

Prediction capacity of the risk score was verified in an independent HCC cohort from the ICGC database. Relationship of the risk score and the prognosis of HCC patients was validated by the Kaplan-Meier curves and ROC curves (two-sided, *P* < 0.05 indicates a statistically significant difference).

### 2.8. Risk Score Based on the Three-Gene Signature Is a Prognostic Risk Factor

We used univariate and multivariate Cox regression analyses to assess whether the risk score based on the three-gene signature and clinical features including age, gender, histopathological grade, pathological stage, T stage, and primary malignancy could be used as prognostic risk factors for patients with HCC. We calculated the hazard ratio (HR) and 95% confidence interval (two-sided, *P* < 0.05 indicates a statistically significant difference).

### 2.9. Building a Combined Nomogram for Clinical Practice

Nomogram is an analytical diagram which simplifies a complex statistical model into a simple survival probability prediction method for an individual patient. In the present study, the risk score and pathological stage were included to construct a nomogram to assess survival probabilities for an individual patient with HCC. Subsequently, the ROC calibration curves were used to examine the accuracy of the nomogram. The overlap of the calibration curve and the reference line indicates a good prediction capacity.

### 2.10. Statistical Analysis

Student's *t*-test was used to compare the two sets of quantitative data. Log-rank tests, Kaplan-Meier curve, and ROC curve were used to identify a gene signature for overall survival prediction. All statistical analyses were performed using SPSS 22.0 (IBM Corp., Armonk, NY, USA) software. Data was presented as mean ± standard deviation, and *P* < 0.05 indicates a statistically significant difference.

## 3. Results

### 3.1. Entire Study Process and Summary of Patients' Information

**Figure 1:**
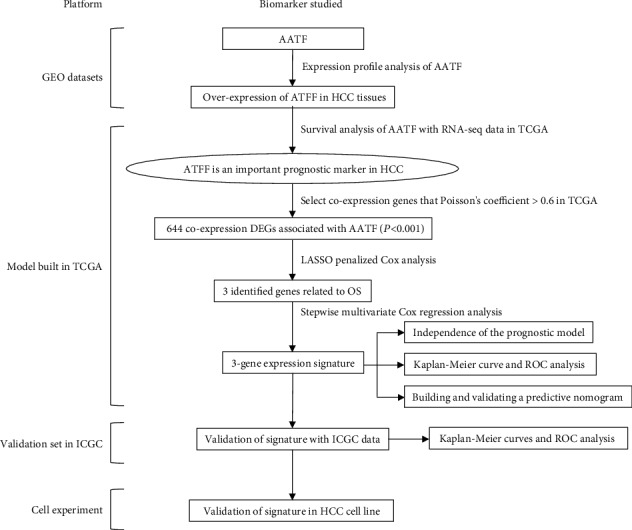
Overall flowchart of this study.

Figure 1 is a flowchart of the entire work in this study. Firstly, we evaluated the prognostic significance of AATF using the data of 2521 HCC patients from the GEO, TCGA, and ICGC databases (GSE22058, *n* = 197; GSE36376, *n* = 433; GSE14520, *n* = 445; GSE54236, *n* = 161; GSE63898, *n* = 396; GSE64041, *n* = 120; GSE76427, *n* = 167; TCGA, *n* = 370; and ICGC, *n* = 232). Comparison of AATF expression between HCC and ANTTs in seven GEO series is shown in Figure S1. Patients' information from the TCGA and ICGC is shown in Table 1.

**Figure 2:**
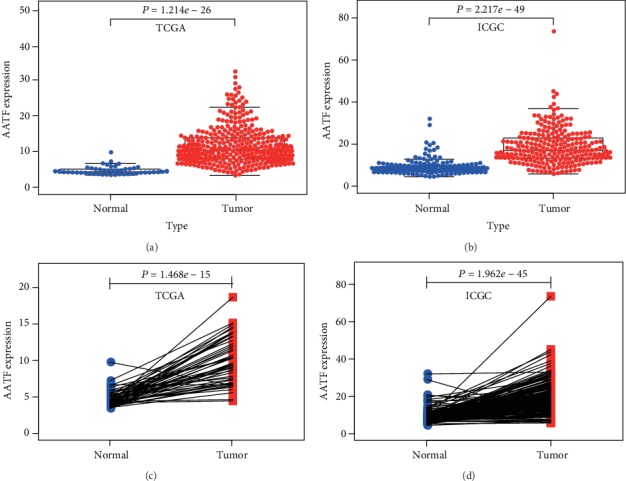
Comparison of AATF mRNA expression between cancer and matched normal liver tissues in the TCGA and ICGC databases. (a, c) AATF mRNA expression levels in the TCGA dataset. (b, d) AATF mRNA expression levels in the ICGC dataset.

**Figure 3:**
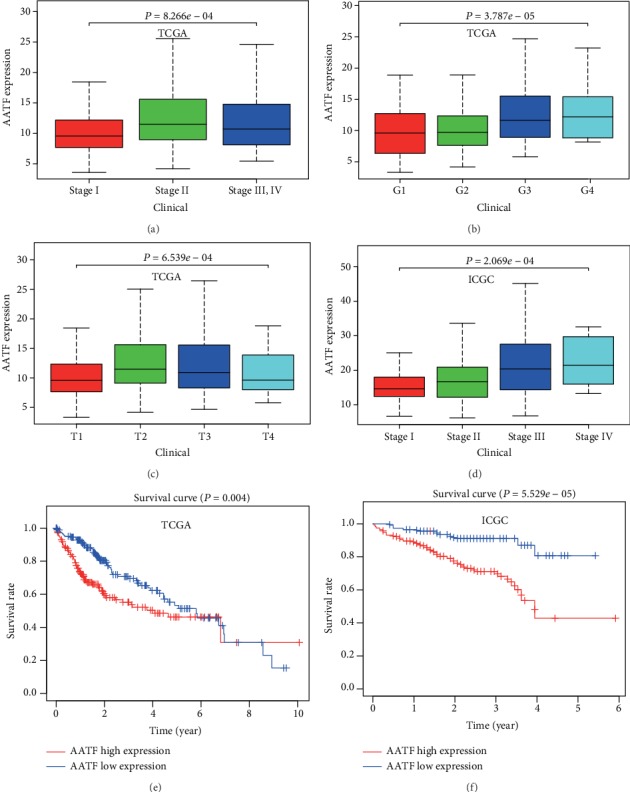
AATF mRNA expression and Kaplan-Meier analysis in the TCGA and ICGC datasets. Comparison of AATF mRNA expression among HCC subgroups divided by TNM stage (a), histopathological grade (b), T stage (c) in the TCGA dataset and by TNM stage in the ICGC dataset (d). Kaplan-Meier analysis was conducted for patients who were assigned to high-and low-risk groups by the AATF expression level in the TCGA (e) and ICGC (f) datasets. Patients with a higher AATF mRNA expression had a poorer prognosis.

We explored the AATF expression profiles in HCC and ANTT. The results of the Wilcoxon signed rank test suggested that AATF expression levels were significantly higher than that in ANTT in the TCGA and ICGC datasets (*P* < 0.05, Figures 2(a)–2(d)). Moreover, AATF expressions were significantly different in HCC tissues at different pathological stages in the TCGA and ICGC datasets (*P* < 0.05, Figures 3(a)–3(d)).

### 3.2. Prognostic Significance of AATF for HCC Patients

To evaluate the prognostic significance of AATF for patients with HCC, we explored the correlation between the AAFT expressions and the overall survival in the TCGA and ICGC datasets. The results suggested that a higher AATF expression level was associated with a poorer prognosis (*P* = 0.004 in TCGA, Figure 3(e); *P* = 5.529*e* − 05 in ICGC, Figure 3(f)).

**Figure 4:**
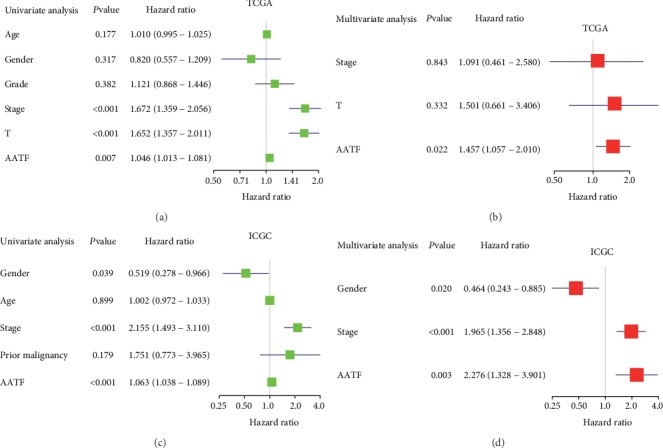
Cox regression analysis showed that AATF mRNA expression was an independent prognostic factor for overall survival of HCC patients. (a) Univariate analysis in the TCGA database. (b) Multivariate analysis in the TCGA database. (c) Univariate analysis in the ICGC database. (d) Multivariate analysis in the ICGC database.

In addition, we explored the relationship between AATF and various clinicopathological factors and the prognosis by Cox regression analysis. The results of both univariate and multivariate Cox regression analyses suggested that a higher AATF expression level was an important risk factor of a poorer prognosis (HR = 1.457, *P* < 0.05 in TCGA, Figures 4(a) and 4(b); HR = 2.276, *P* < 0.01 in ICGC, Figures 4(c) and 4(d)).

### 3.3. Functional Enrichment Analysis of Coexpressed Genes with AATF

We identified 644 coexpressed genes with AATF by the Poisson coefficient (Poisson coefficient > 0.6, *P* < 0.001). The rationale for establishing coexpressed genes associated with AATF is detailed in Table S4. The results of functional enrichment analysis of these 644 coexpressed genes suggested that these genes mainly were enriched in the ncRNA metabolic process, spliceosome, cell cycle, DNA replication, and RNA transport (*P* < 0.05; Figures S2 and S3).

### 3.4. Constructing a Three-Gene Signature for Survival Prediction

**Figure 5:**
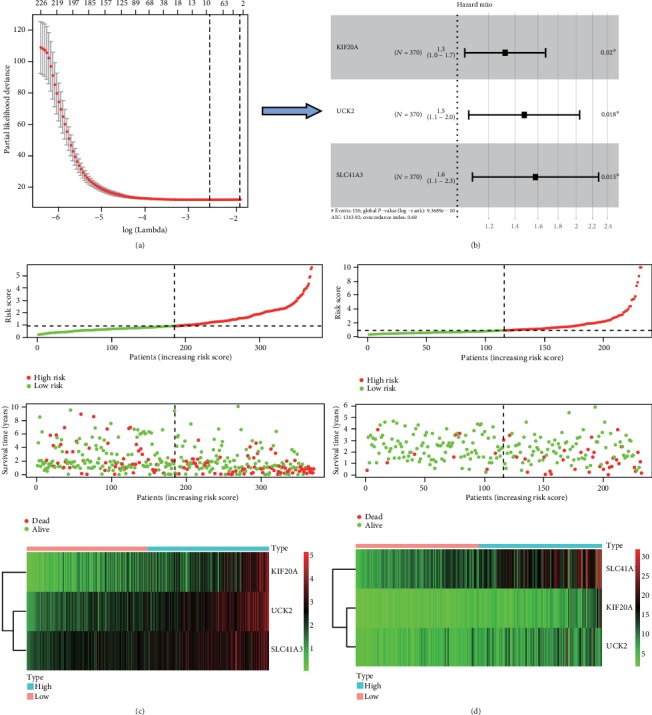
The signature-based risk score is a promising marker in the training and validation cohorts. (a, b) The process of building the signature including 3 genes which were most correlated with overall survival in the training dataset. The hazard ratios (HR) and 95% confidence intervals (CI) were calculated by univariate Cox regression, and the coefficients calculated by multivariate LASSO Cox regression. (c, d) Risk score distribution, survival overview, and heatmap for patients assigned to high-and low-risk groups based on the risk score in the TCGA and ICGC datasets.

A total of 644 coexpressed genes with AATF were used to establish a three-gene signature for survival prediction (Figure 5(a)). We took the TCGA dataset as a training dataset. The LASSO Cox regression was applied to identify stable biomarkers from candidate genes with respect to overall survival. Specifically, we forced the absolute values of the regression coefficient to be less than a fixed value and thus reduced the number of coefficients to zero. Then, the relative regression coefficients were used to identify the most stable markers with prognostic value. Meanwhile, we applied cross-validation to avoid overfitting of the LASSO Cox model. Finally, three key genes including KIF20A, UCK2, and SLC41A3 were identified (HR = 1.3, 1.5, and 1.6, respectively, *P* < 0.05; Figure 5(b)).

Subsequently, we constructed a prognostic model based on KIF20A, UCK2, and SLC41A3 with minimum standards. Firstly, we calculated the risk score of each HCC patient in the training dataset by the LASSO algorithm. Then, we divided 370 HCC patients with follow-up information into high- (*n* = 185) and low-risk groups (*n* = 185) according to the median risk score of all HCC patients in the training dataset. Survival overview of the training dataset is shown in Figure 5(c). It was suggested that the expression levels of KIF20A, UCK2, and SLC41A3 was significantly higher in the high-risk group that in the low-risk group. Next, we took the ICGC as a validation dataset. Similarly, we calculated the risk score of each HCC patient in the validation dataset by using the same formulas used in the training set. According to the median risk score of all HCC patients as the cut-off value, we divided 232 HCC patients of the validation dataset into a high-risk group (*n* = 131) and a low-risk group (*n* = 131). The results also suggested that the expression levels of KIF20A, UCK2, and SLC41A3 were significantly higher in the high-risk group that in the low-risk group (Figure 5(d)).

### 3.5. Biomarker Performance of KIF20A, UCK2, and SLC41A3

**Figure 6:**
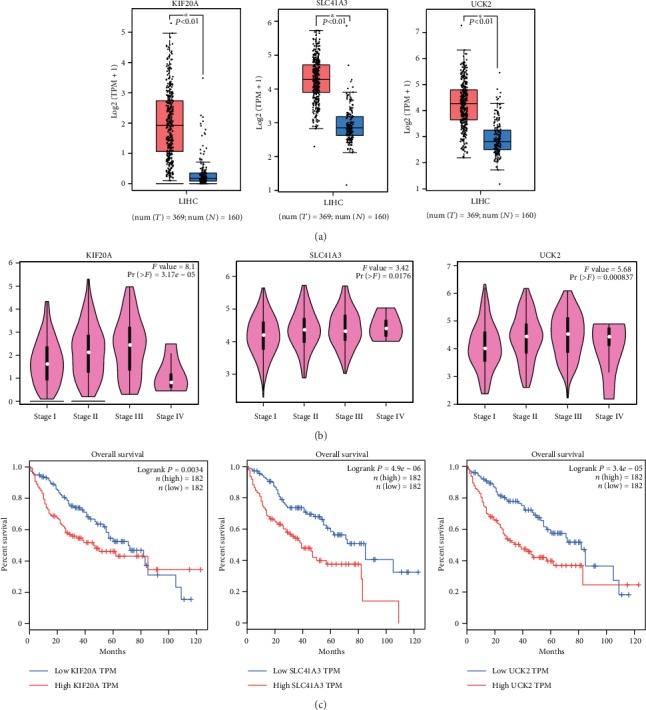
The prognostic value of KIF20A, SLC41A3, and UCK2. (a) Comparison of AATF expression between HCC and ANTT. (b) Comparison of AATF expression among different pathological stages. (c) Relationship between the expressions of KIF20A, SLC41A3, and UCK2 and the prognosis of HCC patients in the TCGA dataset. *P* value was calculated using the log-rank test and shown on the top right corner. ANTT: adjacent nontumor tissues.

In order to verify the biomarker performance of KIF20A, UCK2, and SLC41A3, we examined the expressions of these three genes in the GEPIA database. As shown in Figure 6(a), the results of the one-way ANOVA test showed that the expression levels of KIF20A, UCK2, and SLC41A3 in HCC were significantly higher than those in ANTTs. Moreover, AATF expressions were significantly different in HCC tissues at different pathological stages in the GEPIA database (*P* < 0.05, Figure 6(b)).

In addition, we divided the patients into high- and low-mRNA expression groups according to the median value of KIF20A, UCK2, and SLC41A3 expressions, respectively. Then, we compared the overall survival between the high- and low-mRNA expression groups. The results suggested that a higher mRNA expression was associated with a poorer prognosis for all the three genes (*P* = 0.0034, 4.9*e*-06 and *P* = 3.4*e* − 05, respectively; Figure 6(c)).

Furthermore, we validated the expressions of KIF20A, UCK2, and SLC41A3 in the GEO database (GSE22058, GSE25097, GSE36376, GSE14520, GSE10143, GSE46444, GSE54236, GSE63898, GSE64041, and GSE76427). The results also suggested that a higher mRNA expression was associated with a poorer prognosis for all the three genes (Tables S2, S3, and S4).

In addition, we divided the HCC patients of the TCGA dataset into high- and low-risk groups by the risk score and used GSEA to identify the pathways in which genes of the high-risk group enriched. The results suggested that the genes of the high-risk group enriched in the cell cycle, the p53 signaling pathway, pathways in cancer, the Notch signaling pathway, the WNT signaling pathway, and the TGF-beta signaling pathway increased (Figure S4).

### 3.6. Validating the Biomarker Performance of KIF20A, UCK2, and SLC41A3 by RT-qPCR in an HCC Cell Line

**Figure 7:**
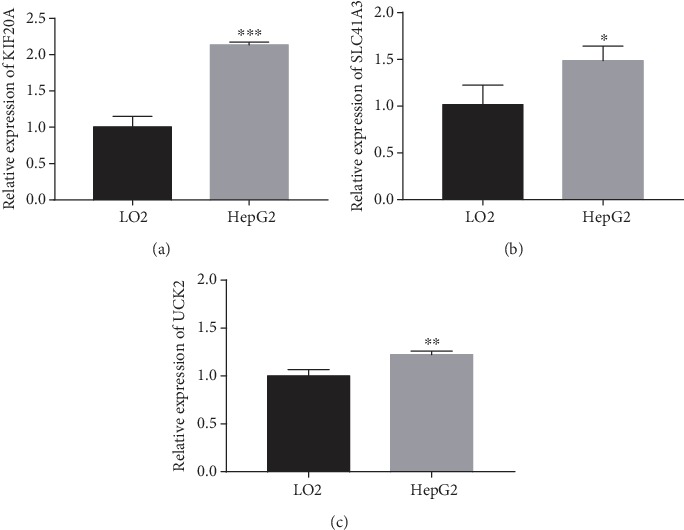
Comparison of mRNA expression between an HCC cell line (HepG2) and normal a liver cell line (LO2) on KIF20A, SLC41A3, and UCK2. (a) KIF20A mRNA expressions in two cell lines. (b) SLC41A3 mRNA expressions in two cell lines. (c) UCK2 mRNA expressions in two cell lines. ^∗^*P* < 0.05; ^∗∗^*P* < 0.01; ^∗∗∗^*P* < 0.001.

We verified the biomarker performance of KIF20A, UCK2, and SLC41A3 in vitro. The results of RT-qPCR suggested that the expression levels of KIF20A, UCK2, and SLC41A3 were significantly higher in HepG2 cells than those in LO2 cells (*P* < 0.05, Figure 7).

### 3.7. Kaplan-Meier and ROC Curves of the Three-Gene Signature

**Figure 8:**
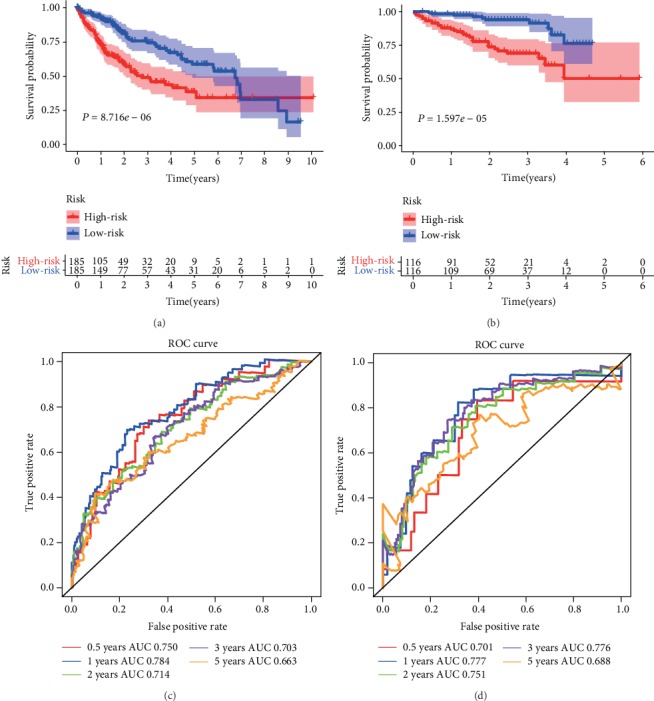
Survival analysis of the three-gene signature in training and validation datasets. Survival possibility was estimated using the Kaplan-Meier method for HCC patients assigned to high-and low-risk groups by the risk score in the TCGA (a) and ICGC (b) datasets. Patients with a higher risk score had a poorer prognosis in the training and validation datasets. The ROC curve was used to verify the predictive efficiency of the risk score in the TCGA (c) and ICGC (d) datasets.

The Kaplan-Meier curve was used to evaluate the prognostic significance of the three-gene signature. Moreover, the ROC curve was used to assess the specificity and sensitivity of the three-gene signature. Taking the median risk score as a cut-off value, we divided the HCC patients into high- and low-risk groups. The results suggested that there was a significant difference in the overall survival between the high-risk and low-risk groups (*P* = 8.716*e* − 06 in TCGA, Figure 8(a); *P* = 1.597*e* − 05 in ICGC, Figure 8(b)). As shown in Figure 8(c), AUC at 1 year was 0.784, at 2 years 0.714, at 3 years 0.703, and at 5 years 0.663 in the TCGA dataset. Meanwhile, as shown in Figure 8(d), the survival rate at 1 year was 0.777, at 2 years 0.751, at 3 years 0.776, and at 5 years 0.668 in the TCGA dataset. The above results confirmed that the three-gene signature can be used as a reliable predictor of overall survival in patients with HCC.

### 3.8. Kaplan-Meier Survival Analysis in Various Pathological Subgroups

**Figure 9:**
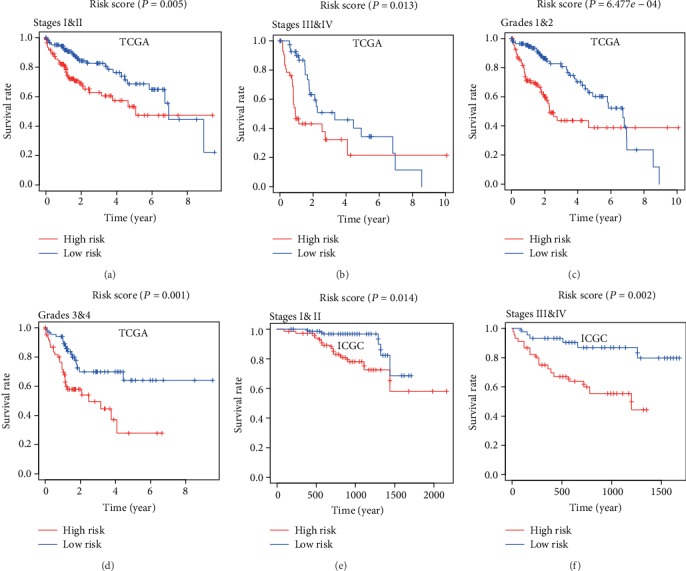
Survival analysis of the risk score in different pathological subgroups of HCC patients. The three-gene based risk score is a promising marker for overall survival in pathological subgroups of stages I and II (a), stages III and IV (b), grades l and 2 (c), grades 3 and 4 (d) in the TCGA dataset and stages I and II (e), stages III and IV (F) in the ICGC dataset.

As shown in Figures 9(a)–9(d), survival analysis by Kaplan-Meier analysis revealed a significant shorter survival in the subgroups of TNM stages I and II HCC with the high-risk group (*P* = 0.005), in TNM stages III and IV HCC with the high-risk group (*P* = 0.013), in grades 1 and 2 HCC with the high-risk group (*P* = 6.477*e* − 04), and in grade 3 and 4 HCC with the high-risk group (*P* = 0.001) in the TCGA dataset. Moreover, as shown in Figures 9(e) and 9(f), survival analysis by Kaplan-Meier analysis also revealed a significant shorter survival in the subgroups of TNM stages I and II HCC with the high-risk group (*P* = 0.014) and in TNM stages III and IV HCC with the high-risk group (*P* = 0.002) in the ICGC dataset. These results suggested that the risk score based on the three-gene gesture had prognostic significance in various subgroups.

### 3.9. The Risk Score Based on the Three-Gene Signature is a Prognostic Risk Factor for HCC Patients

**Figure 10:**
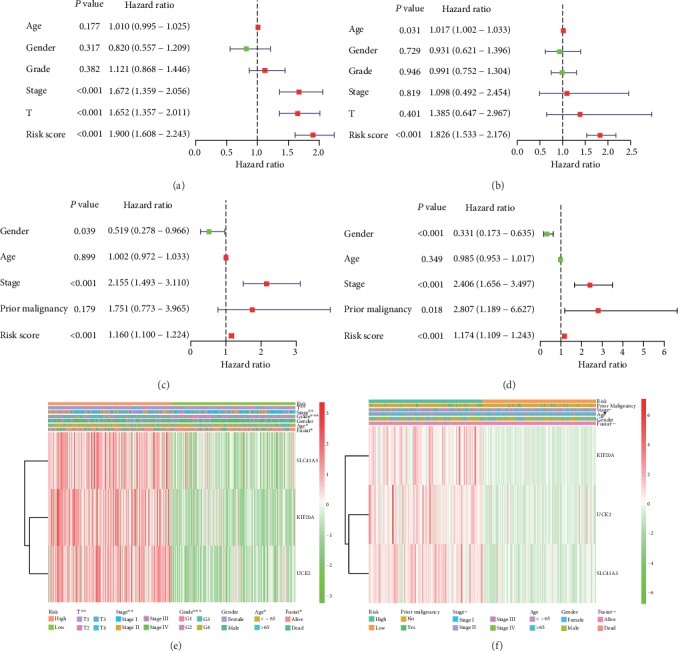
Cox regression analysis of the association between the risk score and clinicopathological features and overall survival. Univariate/multivariate Cox regression analyses of the association between the risk score and clinicopathological factors and overall survival of patients in the TCGA (a, b) and ICGC (c, d) datasets. The heatmaps reveal the association between the risk score and clinicopathological features in the TCGA (e) and ICGC (f). A higher risk score was associated with a higher expression level of SLC41A3, KIF20A, and UCK2. ^∗^*P* < 0.05; ^∗∗^*P* < 0.01; ^∗∗∗^*P* < 0.001.

We used univariate and multivariate Cox regression analyses to assess whether the risk score based on the three-gene signature could be used as a prognostic risk factor for HCC patients. In the TCGA dataset, results of univariate Cox regression indicated that the risk score, TNM stage, and T stage were associated with overall survival in HCC (*P* < 0.001, Figure 10(a)), while results of multivariate Cox regression analysis indicated that only the risk score was associated with overall survival in HCC (*P* < 0.001, Figure 10(b)). In addition, in the ICGC dataset, results of univariate Cox analysis indicated that the risk score, gender, and TNM stage were associated with overall prognosis (*P* < 0.05, Figure 10(c)), while results of multivariate Cox regression analysis indicated that the risk score, previous malignancy, TNM stage, and gender were associated with overall prognosis (*P* < 0.05, Figure 10(d)).

Meanwhile, the heatmap shows the relationship between the risk score and clinical or pathological factors. As shown in Figure 10(e), in the TCGA dataset, there was a significant difference in the risk score between different groups divided by clinical or pathological factors such as age (*P* < 0.05), survival condition (*P* < 0.05), TNM stage (*P* < 0.01), T stage (*P* < 0.01), and grade (*P* < 0.001). Meanwhile, as shown in Figure 10(f), in the ICGC dataset, there was a significant difference in the risk score between the different groups divided by clinical or pathological factors such as TNM stage (*P* < 0.01) and survival condition (*P* < 0.001).

### 3.10. Building a Combined Nomogram for Clinical Practice

**Figure 11:**
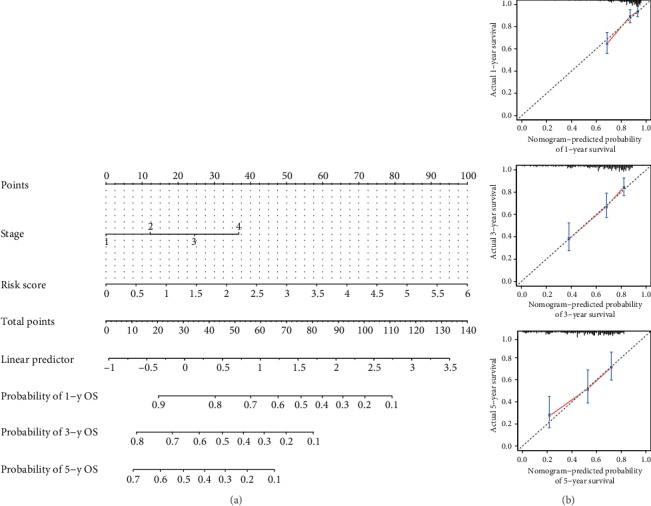
Construction of a nomogram based on the three-gene signature for overall survival prediction. (a) The nomogram combining the risk score and TNM stage. (b) The calibration plots showed that the predicted 1-, 3- and 5-year survival probabilities agreed well with the actual observations.

We built a combined nomogram based on the risk score and TNM stage to for clinical practice (Figure 11(a)). Then, calibration curves were used to validate the prediction capacity of this nomogram (Figure 11(b)). The calibration plots showed that the predicted 1-, 3- and 5-year survival probabilities agreed well with the actual observations.

**Figure 12:**
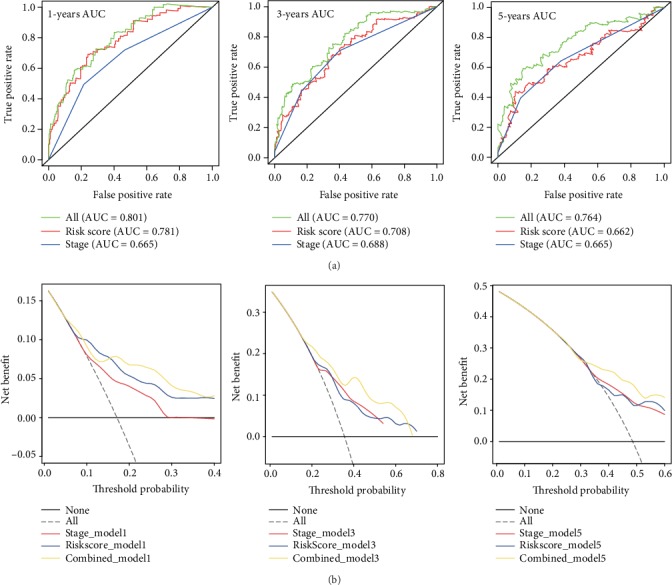
ROC curves of the combined nomogram. (a) ROC curves showed the predictive efficiency of the risk score, TNM stage, and the combined model at 1-year, 3-year, and 5-year survival predictions in the validation dataset. (b) Relations between net benefit and threshold probability at 1-year, 3-year, and 5-year survival predictions.

In addition, ROC curves were used to assess the prediction accuracy of the risk score, TNM stage, and the combined nomogram for 1-, 3- and 5-year survival probabilities. In Figure 12(a), the green, red, and blue solid lines represent the ROC plots of the combined nomogram, the risk score, and the TNM stage, respectively. The AUCs of the combined nomogram were the largest of the three models (AUC = 0.801 at 1 year, 0.770 at 3 year, and 0.764 at 5 year). Meanwhile, Figure 12(b) indicated greater net benefit for the combined model than for either risk score or TNM stage alone.

## 4. Discussion

HCC is one of the most life-threatening malignancies worldwide. Accurate survival prediction can help to develop a personalized treatment plan for patients with HCC. Nomogram is a new tool to predict the prognosis of patients with cancers.

In this study, we extracted gene expression data and clinical information from three public databases including the GEO, TCGA, and ICGC. AATF was used as a key gene to identify important genes contributing to the occurrence and development of HCC. Genes coexpressed with AATF in HCC were screened out by the Poisson correlation coefficient. A three-gene signature including KIF20A, UCK2, and SLC41A3 was identified by univariate and LASSO regression analyses.

KIF20A is a gene related to pathways including vesicle-mediated transport and innate immune system. Gene Ontology (GO) annotations related to this gene include protein kinase binding and ATPase activity. UCK2 is related to metabolism of nucleotides and fluoropyrimidine activity. GO annotations related to this gene include kinase activity and dephospho-CoA kinase activity. SLC41A3 is related to cation transmembrane transporter activity. The GO annotations of these 3 genes suggested that abnormalities in DNA replication and RNA transport play an important role in the occurrence and development of HCC.

We validated the prognostic significance of the risk score based on the three-gene signature by the Kaplan-Meier analysis and ROC curves. It was proved that a higher risk score was associated with a poorer prognosis in patients with HCC both in the TCGA and ICGC datasets. Therefore, the risk score is a promising prognostic risk factor which predicts the overall survival of HCC patients well. Moreover, we established a combined nomogram that could predict survival probabilities for an individual HCC patient. Then, the calibration plots and the ROC curves were used to verify the survival prediction accuracy of the nomogram in HCC.

Gene expression signatures have prognostic significance in HCC [10]. It is reported that gene expression signatures can predict overall survival well in HCC [11, 12]. The prognostic value of gene signatures can help to develop individual treatments and improve the prognosis for HCC patients [13, 14]. Meanwhile, gene signatures can provide clues of potential therapeutic targets to improve clinical outcomes of HCC. Many prognostic gene signatures including mRNA, miRNA, or lncRNA were identified in previous studies, and the gene number of these gene signatures may be different [15–23]. In the present study, AATF, a key transcription factor contributing to the occurrence and development of HCC, was used to identify coexpressed genes which may play an important role in the molecular mechanisms in the AATF regulation process of gene transcription [24]. The highlight of our study was that the sample size was big. The mRNA expression data of 2521 HCC patients were downloaded from the GEO, TCGA, and ICGC databases. The three-gene signature built in this study can broaden the scope of gene signatures of HCC. Furthermore, the combined nomogram has a good prognostic significance. The area of ROC that predicts survival was 0.801 at 1-year survival, 0.770 at 3-year survival and 0.764 at 5-year survival.

To our knowledge, this three-gene signature identified in our study has not been reported before. Besides, we verified the overexpression of KIF20A, UCK2, and SLC41A3 in an HCC cell line, which made the reliability of our model more convincing. Furthermore, it had been proved that the combined nomogram based on the three-gene signature can predict overall survival well by the calibration plots and the ROC curves.

However, this study has certain limitations. For example, the expression of KIF20A, UCK2, and SLC41A3 should be determined in a larger cohort of HCC cell lines with different phenotypes to demonstrate a putative relationship with tumor aggressiveness. So we will carry out this experiment when conditions are available in the future. In addition, the clinical heterogeneity of HCC may affect the prediction accuracy of this nomogram. Therefore, combining this nomogram with other routine assessment methods according to the actual situation of patient can better predict the overall survival of HCC.

Taken together, we established a prognostic three-gene signature of HCC patients in this study. The nomogram based on the risk score calculated by the three-gene signature was validated to be a reliable tool in predicting survival possibility for an individual patient with HCC. In addition, this three-gene signature may be a potential therapeutic target which can provide clues for exploring the regulatory mechanisms of AATF in HCC.

## Figures and Tables

**Table 1:** Patients' information of this study from the ICGC and TCGA databases.

Clinical characteristics			*N*	(%)
ICGC	Survival status	Survival	189	81.47
		Death	43	18.53
	Age	≤65 years	90	38.79
		>65 years	142	61.21
	Gender	Male	171	73.71
		Female	61	26.29
	Stage	I	36	15.52
		II	106	45.69
		III	71	30.6
		IV	19	8.19
	Prior malignancy	No	202	87.07
		Yes	30	12.93
Total			232	

TCGA	Survival status	Survival	244	65.95
		Death	126	34.05
	Age	≤65 years	232	62.7
		>65 years	138	37.3
	Gender	Male	249	67.3
		Female	121	32.7
	Histological grade	G1	55	14.86
		G2	177	47.84
		G3	121	32.7
		G4	12	3.24
	Stage	I	171	46.22
		II	85	22.97
		III	85	22.97
		IV	5	1.35
	T classification	T1	181	48.92
		T2	93	25.14
		T3	80	21.62
		T4	13	3.51
		TX	1	0.27
	M classification	M0	266	71.89
		M1	4	1.08
		MX	100	27.03
	N classification	N0	252	68.11
		N1	4	1.08
		NX	113	30.54
Total			370	

## Data Availability

The datasets analyzed in this study are available the GEO (https://www.ncbi.nlm.nih.gov/geo/), the TCGA repository (http://cancergenome.nih.gov/), and the ICGC (https://icgc.org/).

## Supplementary Materials

Supplementary Materials Figure S1: comparison of AATF expression between HCC and ANTTs in seven GEO series. ANTTs: adjacent non-tumor tissues. Figure S2: GO enrichment analysis of co-expressed genes with AATF. GO analysis revealed the most significant biological process, molecular function and cell cellular in HCC. Figure S3: KEGG analysis of co-expressed genes with AATF. KEGG analysis revealed the most important pathways in HCC. Figure S4: GSEA shows upregulation of signaling response genes in HCC. NES, normalized enrichment score. Values are row-scaled to show relative expression. Blue and red are low and high levels, respectively. Table S1: expression analysis of KIF20A in GEO. Table S2: expression analysis of UCK2 in GEO. Table S3: expression analysis of SLC41A3 in GEO. Table 4S: Co-expressed genes with AATF (Poisson coefficient>0.6).

## References

[1] The Cancer Genome Atlas Research Network (2017). Comprehensive and integrative genomic characterization of hepatocellular carcinoma. *Cell*.

[2] Budhu A., Forgues M., Ye Q. H. (2006). Prediction of venous metastases, recurrence, and prognosis in hepatocellular carcinoma based on a unique immune response signature of the liver microenvironment. *Cancer Cell*.

[3] Kim S. M., Leem S. H., Chu I. S. (2012). Sixty-five gene-based risk score classifier predicts overall survival in hepatocellular carcinoma. *Hepatology*.

[4] Kumar D. P., Santhekadur P. K., Seneshaw M., Mirshahi F., Uram-Tuculescu C., Sanyal A. J. (2019). A regulatory role of apoptosis antagonizing transcription factor in the pathogenesis of nonalcoholic fatty liver disease and hepatocellular carcinoma. *Hepatology*.

[5] Yamashita T., Forgues M., Wang W. (2008). EpCAM and alpha-fetoprotein expression defines novel prognostic subtypes of hepatocellular carcinoma. *Cancer Research*.

[6] Woo H. G., Park E. S., Cheon J. H. (2008). Gene expression-based recurrence prediction of hepatitis B virus-related human hepatocellular carcinoma. *Clinical Cancer Research*.

[7] Coulouarn C., Factor V. M., Thorgeirsson S. S. (2008). Transforming growth factor-beta gene expression signature in mouse hepatocytes predicts clinical outcome in human cancer. *Hepatology*.

[8] Long J., Zhang L., Wan X. (2018). A four-gene-based prognostic model predicts overall survival in patients with hepatocellular carcinoma. *Journal of Cellular and Molecular Medicine*.

[9] Yang L., Xu Y., Yan Y. (2019). Common nevus and skin cutaneous melanoma: prognostic genes identified by gene co-expression network analysis. *Genes*.

[10] Makowska Z., Boldanova T., Adametz D. (2016). Gene expression analysis of biopsy samples reveals critical limitations of transcriptome-based molecular classifications of hepatocellular carcinoma. *The Journal of Pathology Clinical Research*.

[11] Gu J., Zhang X., Miao R. (2018). A three-long non-coding RNA-expression-based risk score system can better predict both overall and recurrence-free survival in patients with small hepatocellular carcinoma. *Aging*.

[12] Zheng Y., Liu Y., Zhao S. (2018). Large-scale analysis reveals a novel risk score to predict overall survival in hepatocellular carcinoma. *Cancer Management and Research*.

[13] Ao L., Song X., Li X. (2016). An individualized prognostic signature and multi‑omics distinction for early stage hepatocellular carcinoma patients with surgical resection. *Oncotarget*.

[14] Gillet J. P., Andersen J. B., Madigan J. P. (2016). A gene expression signature associated with overall survival in patients with hepatocellular carcinoma suggests a new treatment strategy. *Molecular Pharmacology*.

[15] Li C., Li J., Xue K. (2019). MicroRNA-143-3p promotes human cardiac fibrosis via targeting sprouty3 after myocardial infarction. *Journal of Molecular and Cellular Cardiology*.

[16] Wang Z., Teng D., Li Y., Hu Z., Liu L., Zheng H. (2018). A six-gene-based prognostic signature for hepatocellular carcinoma overall survival prediction. *Life Sciences*.

[17] Liu G., Wang H., Fu J. D., Liu J. Y., Yan A. G., Guan Y. Y. (2017). A five-miRNA expression signature predicts survival in hepatocellular carcinoma. *APMIS*.

[18] Liao X., Zhu G., Huang R. (2018). Identification of potential prognostic microRNA biomarkers for predicting survival in patients with hepatocellular carcinoma. *Cancer Management and Research*.

[19] Lu M., Kong X., Wang H., Huang G., Ye C., He Z. (2017). A novel microRNAs expression signature for hepatocellular carcinoma diagnosis and prognosis. *Oncotarget*.

[20] Yan J., Zhou C., Guo K., Li Q., Wang Z. (2019). A novel seven-lncRNA signature for prognosis prediction in hepatocellular carcinoma. *Journal of Cellular Biochemistry*.

[21] Zhao Q. J., Zhang J., Xu L., Liu F. F. (2018). Identification of a five-long non-coding RNA signature to improve the prognosis prediction for patients with hepatocellular carcinoma. *World Journal of Gastroenterology*.

[22] Wang Y., Ruan Z., Yu S. (2019). A four-methylated mRNA signature-based risk score system predicts survival in patients with hepatocellular carcinoma. *Aging*.

[23] Liu S., Miao C., Liu J., Wang C. C., Lu X. J. (2018). Four differentially methylated gene pairs to predict the prognosis for early stage hepatocellular carcinoma patients. *Journal of Cellular Physiology*.

[24] Chen P., Wang F., Feng J. (2017). Co-expression network analysis identified six hub genes in association with metastasis risk and prognosis in hepatocellular carcinoma. *Oncotarget*.

