# Supplementary material for: A Nomogram Based on a Three-Gene Signature Derived from AATF Coexpressed Genes Predicts Overall Survival of Hepatocellular Carcinoma Patients
Source: Biomed Res Int. 2020 Apr 22;2020:7310768. doi: 10.1155/2020/7310768 (PMC7195644; doi:10.1155/2020/7310768)
Supplement: Supplementary Materials — Figure S1: comparison of AATF expression between HCC and ANTTs in seven GEO series. ANTTs: adjacent non-tumor tissues. Figure S2: GO enrichment analysis of co-expressed genes with AATF. GO analysis revealed the most significant biological process, molecular function and cell cellular in HCC. Figure S3: KEGG analysis of co-expressed genes with AATF. KEGG analysis revealed the most important pathways in HCC. Figure S4: GSEA shows upregulation of signaling response genes in HCC. NES, normalized enrichment score. Values are row-scaled to show relative expression. Blue and red are low and high levels, respectively. Table S1: expression analysis of KIF20A in GEO. Table S2: expression analysis of UCK2 in GEO. Table S3: expression analysis of SLC41A3 in GEO. Table 4S: Co-expressed genes with AATF (Poisson coefficient>0.6). [file 7310768.f1.pdf]

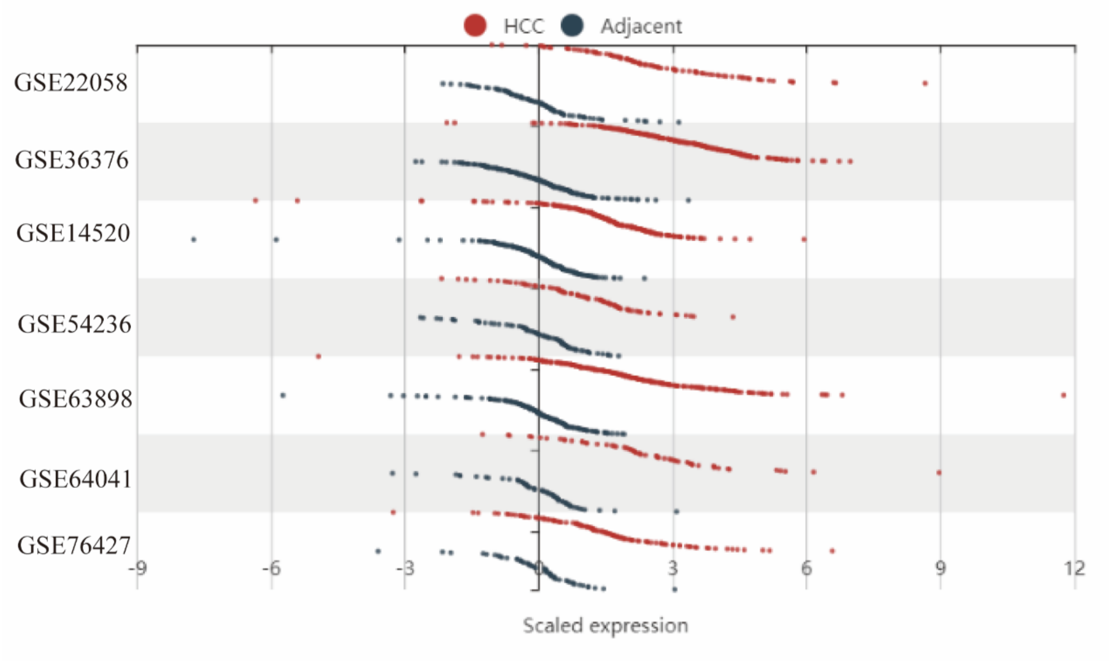

Figure S1. Comparison of AATF expression between HCC and ANTTs in seven GEO series. ANTTs: adjacent non-tumor tissues.

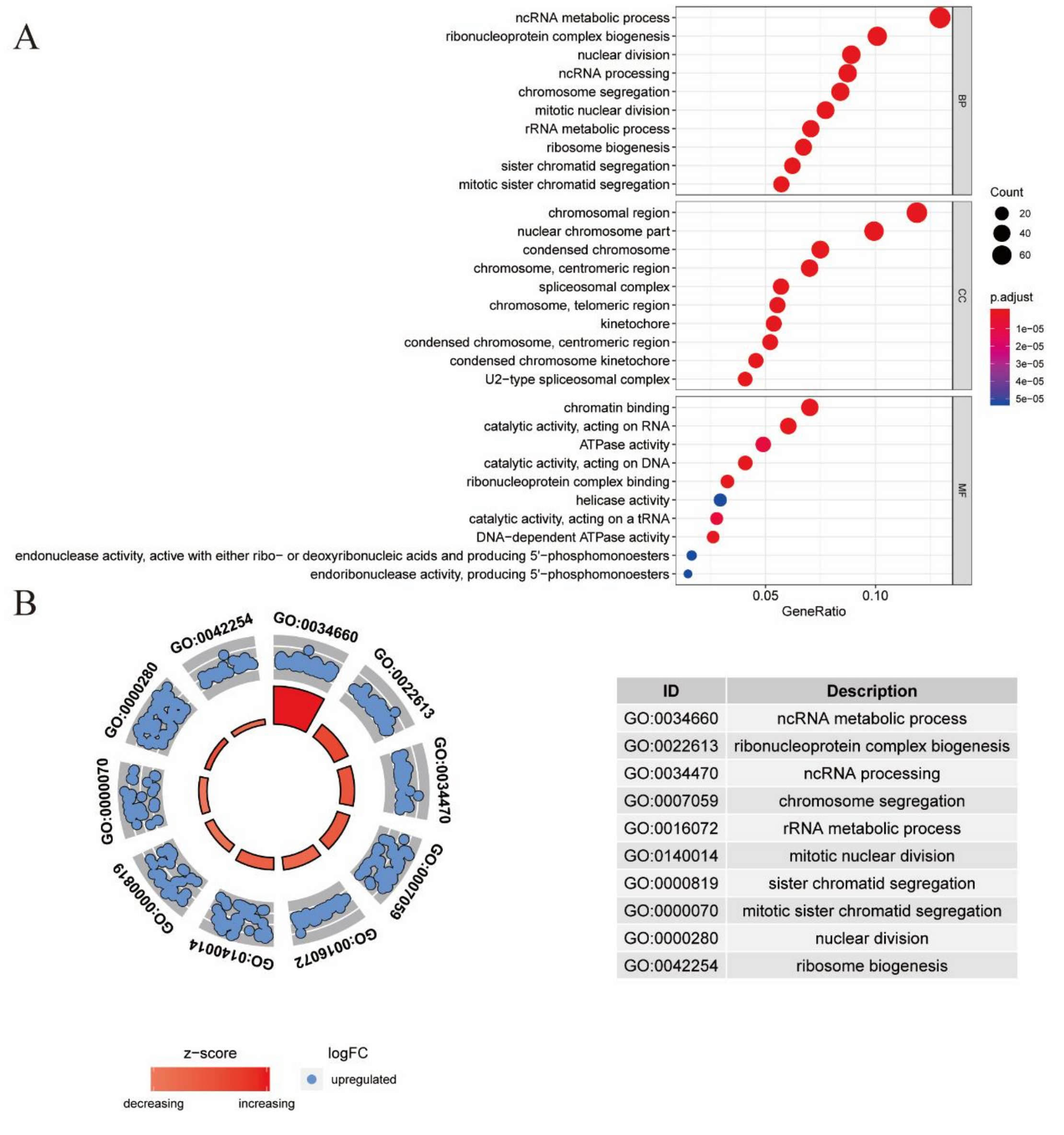

Figure S2. GO enrichment analysis of co-expressed genes with AATF. GO analysis revealed the most significant biological process, molecular function and cell cellular in HCC.

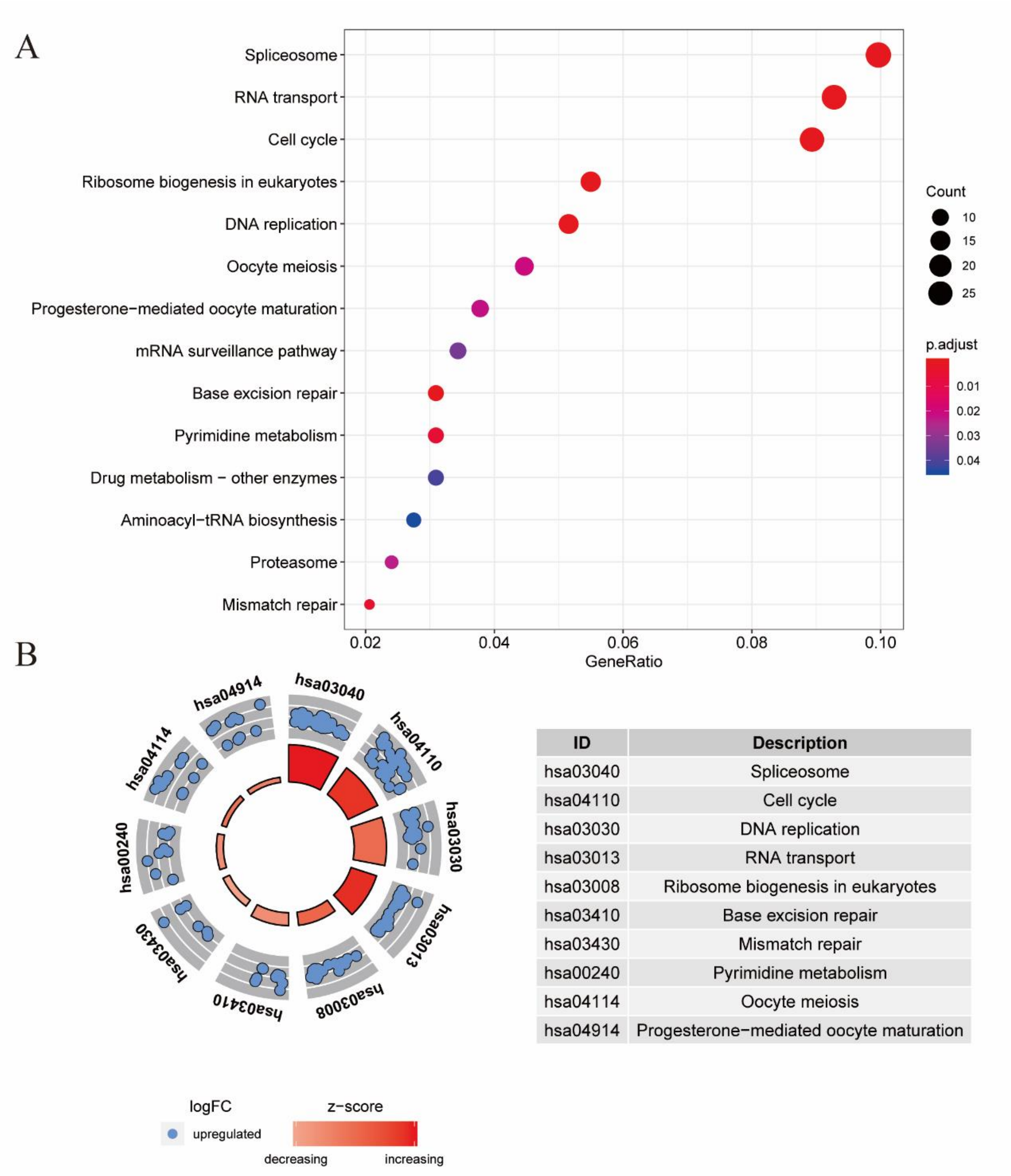

Figure S3. KEGG analysis of co-expressed genes with AATF. KEGG analysis revealed the most important pathways in HCC.

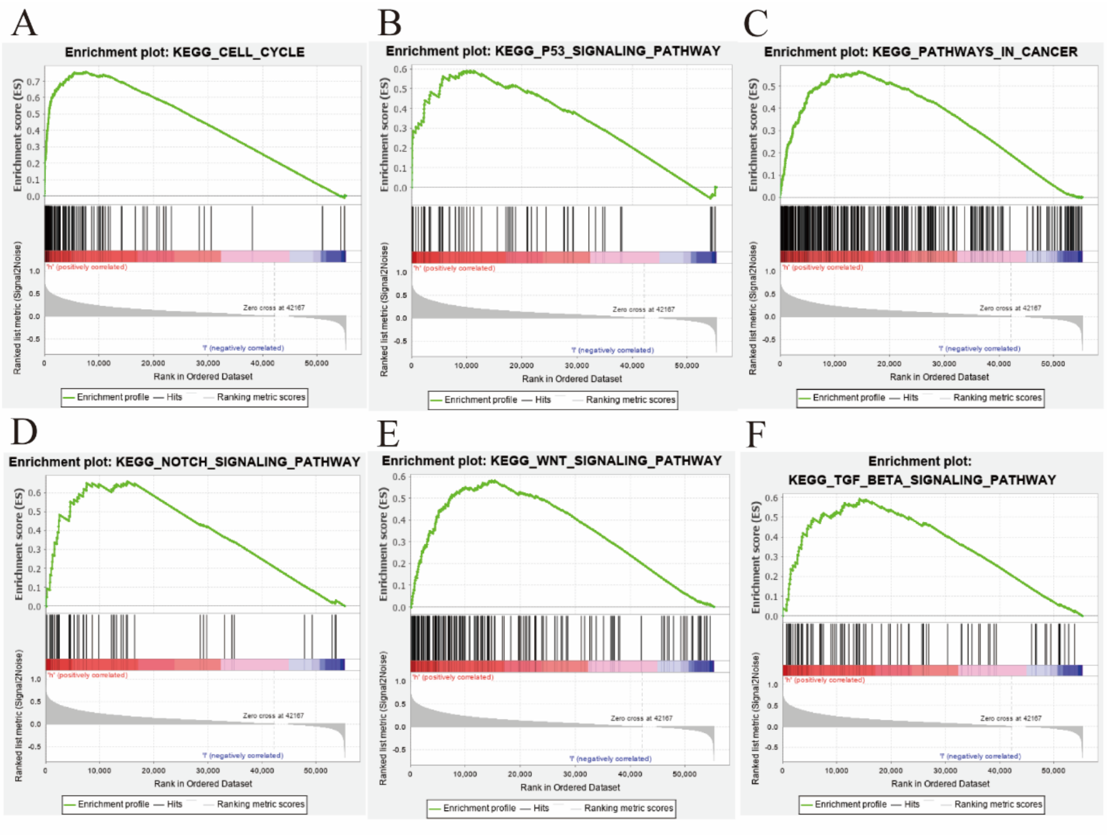

Figure S4. GSEA shows upregulation of signaling response genes in HCC. NES, normalized enrichment score. Values are row-scaled to show relative expression. Blue and red are low and high levels, respectively.

Table S1 Expression analysis of KIF20A in GEO

Dataset	P-value	Type	Num	Mean	STD	IQR
GSE22058	1.63E-55	HCC	100	8.772	1.14	1.582
		Adjacent	97	5.497	0.8598	1.266
GSE25097	1.05E-50	HCC	268	1.026	0.8076	0.9293
		Adjacent	243	0.09964	0.08843	0.067
GSE36376	2.48E-66	HCC	240	7.295	0.5396	0.7995
		Adjacent	193	6.46	0.1381	0.2076
GSE14520	1.18E-67	HCC	225	5.198	1.021	1.627
		Adjacent	220	3.501	0.2568	0.2612
GSE46444	0.02358	HCC	88	6.747	1.196	1.751
		Adjacent	48	7.42	1.81	2.79
GSE54236	4.32E-17	HCC	81	6.363	1.466	1.915
		Adjacent	80	4.492	0.9458	1.026
GSE63898	3.05E-52	HCC	228	5.572	0.9925	1.327
		Adjacent	168	4.255	0.2321	0.253
GSE64041	2.06E-13	HCC	60	6.098	1.056	1.416
		Adjacent	60	4.795	0.4261	0.3502
GSE64041	2.55E-29	HCC	115	8.163	0.702	0.99
		Adjacent	52	7.071	0.3201	0.305

Table S2 Expression analysis of UCK2 in GEO

Dataset	P-value	Type	Num	Mean	STD	IQR
GSE22058	4.80E-14	HCC	100	8.402	0.6534	0.839
		Adjacent	97	7.774	0.3814	0.4813
GSE25097	4.17E-34	HCC	268	2.216	1.215	1.486
		Adjacent	243	1.131	0.4475	0.5868
GSE36376	1.14E-70	HCC	240	7.284	0.6465	0.9894
		Adjacent	193	6.231	0.2423	0.3289
GSE14520	5.09E-73	HCC	225	5.536	0.8175	1.171
		Adjacent	220	4.097	0.3763	0.3883
GSE10143	5.76E-05	HCC	80	12.53	1.041	1.153
		Adjacent	82	11.86	1.025	1.18
GSE46444	0.009643	HCC	88	7.556	1.417	2.627
		Adjacent	48	6.891	1.397	2.592
GSE54236	2.96E-14	HCC	81	10.1	0.8238	1.006
		Adjacent	80	9.199	0.4522	0.4887
GSE63898	7.91E-37	HCC	228	5.958	0.4244	0.5974
		Adjacent	168	5.456	0.2818	0.3907
GSE64041	2.57E-13	HCC	60	8.672	0.507	0.527
		Adjacent	60	8.045	0.2318	0.1702
GSE76427	2.56E-15	HCC	115	8.334	0.4967	0.745
		Adjacent	52	7.702	0.3795	0.375

Table S3 Expression analysis of SLC41A3 in GEO

Dataset	P-value	Type	Num	Mean	STD	IQR
GSE22058	1.56E-23	HCC	100	9.693	0.5147	0.6715
		Adjacent	97	8.99	0.2932	0.3328
GSE25097	9.26E-49	HCC	268	1.612	0.6054	0.846
		Adjacent	243	0.9248	0.2248	0.2615
GSE36376	1.16E-89	HCC	240	8.563	0.505	0.6518
		Adjacent	193	7.498	0.3437	0.3744
GSE14520	1.27E-30	HCC	225	5.514	0.4485	0.622
		Adjacent	220	5.067	0.2857	0.3215
GSE54236	7.04E-06	HCC	81	11.42	0.4316	0.574
		Adjacent	80	11.14	0.3226	0.3162
GSE63898	2.84E-41	HCC	228	5.997	0.4727	0.5992
		Adjacent	168	5.42	0.2742	0.3484
GSE64041	5.47E-09	HCC	60	8.318	0.3457	0.4254
		Adjacent	60	7.986	0.2043	0.2261
GSE76427	1.89E-13	HCC	115	9.338	0.4652	0.585
		Adjacent	52	8.783	0.3697	0.3575

Table 4S Co-expressed genes with AATF (Poisson coefficient>0.6)

Gene1	Gene2	Gene2 Type	Coefficient	P-value
AATF	SLC26A6	protein_coding	0.637	1.12365E-49
AATF	CDC37	protein_coding	0.668	4.41942E-56
AATF	CCDC97	protein_coding	0.646	2.18095E-51
AATF	SPSB2	protein_coding	0.659	4.61814E-54
AATF	TIMM9	protein_coding	0.606	8.12944E-44
AATF	C12orf73	protein_coding	0.658	5.25521E-54
AATF	BOD1	protein_coding	0.669	3.10064E-56
AATF	LARS	protein_coding	0.606	6.18118E-44
AATF	EXOSC2	protein_coding	0.616	1.17434E-45
AATF	DDX51	protein_coding	0.66	2.67048E-54
AATF	BUD13	protein_coding	0.645	3.66119E-51
AATF	NPLOC4	protein_coding	0.654	4.36179E-53
AATF	METTL6	protein_coding	0.671	7.49432E-57
AATF	EXOSC3	protein_coding	0.631	1.57996E-48
AATF	PTRH2	protein_coding	0.629	4.60673E-48
AATF	FBXW9	protein_coding	0.603	2.68261E-43
AATF	LIG1	protein_coding	0.614	2.30339E-45
AATF	TROAP	protein_coding	0.615	2.15235E-45
AATF	CDK1	protein_coding	0.622	7.80771E-47
AATF	CPSF3	protein_coding	0.73	8.33764E-72
AATF	JMJD6	protein_coding	0.737	1.04223E-73
AATF	MMS19	protein_coding	0.624	4.17594E-47
AATF	CDC25C	protein_coding	0.61	1.65197E-44
AATF	NHP2	protein_coding	0.715	1.58876E-67
AATF	SMARCE1	protein_coding	0.643	7.54352E-51
AATF	IP6K1	protein_coding	0.646	1.95779E-51
AATF	TTC27	protein_coding	0.757	6.28658E-80
AATF	H2AFZ	protein_coding	0.664	3.24191E-55
AATF	G6PC3	protein_coding	0.631	1.63467E-48
AATF	ADPRHL2	protein_coding	0.607	5.15097E-44
AATF	COPZ1	protein_coding	0.708	7.35752E-66
AATF	COG1	protein_coding	0.666	1.1072E-55
AATF	RSL1D1	protein_coding	0.604	1.86792E-43
AATF	TK1	protein_coding	0.705	4.58065E-65
AATF	TSEN54	protein_coding	0.751	3.28303E-78
AATF	DAXX	protein_coding	0.7	8.6573E-64
AATF	JPT1	protein_coding	0.705	5.2008E-65
AATF	LYRM4	protein_coding	0.635	2.85889E-49
AATF	DUSP28	protein_coding	0.61	1.23065E-44
AATF	METTL23	protein_coding	0.713	4.24573E-67
AATF	VARS	protein_coding	0.653	7.91353E-53
AATF	NSUN5	protein_coding	0.777	9.1419E-87

AATF	TONSL	protein_coding	0.63	2.79725E-48
AATF	DPH2	protein_coding	0.665	2.24592E-55
AATF	UBE2D2	protein_coding	0.666	1.31187E-55
AATF	RAD51D	protein_coding	0.645	2.49679E-51
AATF	CDC45	protein_coding	0.659	3.931E-54
AATF	LIG3	protein_coding	0.694	2.90485E-62
AATF	FARSA	protein_coding	0.632	1.34417E-48
AATF	FAAP24	protein_coding	0.683	1.74942E-59
AATF	RAD18	protein_coding	0.613	4.83673E-45
AATF	HAX1	protein_coding	0.611	1.00505E-44
AATF	TATDN2	protein_coding	0.625	2.45678E-47
AATF	TEFM	protein_coding	0.614	2.55262E-45
AATF	DNAAF5	protein_coding	0.614	2.92229E-45
AATF	HSPBP1	protein_coding	0.616	1.45274E-45
AATF	DSCC1	protein_coding	0.671	6.76657E-57
AATF	PHB	protein_coding	0.751	3.94001E-78
AATF	RBM42	protein_coding	0.685	5.98842E-60
AATF	RRP1	protein_coding	0.662	7.50297E-55
AATF	TBCB	protein_coding	0.659	4.56798E-54
AATF	DKC1	protein_coding	0.628	6.1316E-48
AATF	EIF6	protein_coding	0.618	4.88714E-46
AATF	KNOP1	protein_coding	0.654	4.99991E-53
AATF	TTC1	protein_coding	0.62	2.40301E-46
AATF	MIF4GD	protein_coding	0.604	1.58997E-43
AATF	SSRP1	protein_coding	0.658	7.63159E-54
AATF	TAF9	protein_coding	0.625	2.47401E-47
AATF	PRPF3	protein_coding	0.656	1.86054E-53
AATF	DXO	protein_coding	0.603	2.02321E-43
AATF	THOC5	protein_coding	0.648	7.98708E-52
AATF	C17orf75	protein_coding	0.679	1.14622E-58
AATF	RFC4	protein_coding	0.697	5.55653E-63
AATF	IFT20	protein_coding	0.609	2.06178E-44
AATF	PSME3	protein_coding	0.692	1.19422E-61
AATF	RAD51	protein_coding	0.662	6.56256E-55
AATF	CDC6	protein_coding	0.636	1.84461E-49
AATF	VPS33B	protein_coding	0.675	9.86333E-58
AATF	SKA3	protein_coding	0.607	5.94199E-44
AATF	ZNF335	protein_coding	0.61	1.20597E-44
AATF	ISG20L2	protein_coding	0.621	1.62206E-46
AATF	ZPR1	protein_coding	0.618	6.26263E-46
AATF	GRK6	protein_coding	0.716	7.91442E-68
AATF	EFNA4	protein_coding	0.678	1.70296E-58
AATF	TIMM17B	protein_coding	0.648	7.42034E-52
AATF	INO80E	protein_coding	0.633	7.95635E-49

AATF	ALKBH4	protein_coding	0.613	4.12101E-45
AATF	H2AFY	protein_coding	0.638	9.69321E-50
AATF	CNOT10	protein_coding	0.632	1.05886E-48
AATF	PDAP1	protein_coding	0.689	6.76981E-61
AATF	WDCP	protein_coding	0.652	1.25814E-52
AATF	SUB1	protein_coding	0.626	1.37502E-47
AATF	BRD9	protein_coding	0.636	2.133E-49
AATF	TJAP1	protein_coding	0.639	3.95201E-50
AATF	UBXN1	protein_coding	0.63	3.22975E-48
AATF	NDRG3	protein_coding	0.685	6.49786E-60
AATF	PRR11	protein_coding	0.629	3.73218E-48
AATF	MORN2	protein_coding	0.613	3.37461E-45
AATF	TRAPPC3	protein_coding	0.62	2.5921E-46
AATF	CERS5	protein_coding	0.681	5.15178E-59
AATF	NME2	protein_coding	0.623	6.91786E-47
AATF	HSPB11	protein_coding	0.666	1.20119E-55
AATF	EZH2	protein_coding	0.636	1.91905E-49
AATF	MXD3	protein_coding	0.663	5.76438E-55
AATF	ZCRB1	protein_coding	0.627	1.10202E-47
AATF	HAUS8	protein_coding	0.615	1.48715E-45
AATF	PPIL1	protein_coding	0.625	2.24418E-47
AATF	FLOT1	protein_coding	0.632	1.27623E-48
AATF	TEDC2	protein_coding	0.641	1.69791E-50
AATF	ANAPC11	protein_coding	0.606	6.74046E-44
AATF	YTHDF1	protein_coding	0.615	1.93328E-45
AATF	RALA	protein_coding	0.625	2.54318E-47
AATF	KDM1A	protein_coding	0.645	2.65497E-51
AATF	GAR1	protein_coding	0.617	9.26416E-46
AATF	EIF3D	protein_coding	0.723	8.03946E-70
AATF	EIPR1	protein_coding	0.637	1.31694E-49
AATF	GRN	protein_coding	0.729	2.20201E-71
AATF	SEC13	protein_coding	0.625	2.26938E-47
AATF	SMARCD1	protein_coding	0.645	3.18854E-51
AATF	WDR70	protein_coding	0.728	2.6623E-71
AATF	TUBG1	protein_coding	0.731	3.90982E-72
AATF	ASNA1	protein_coding	0.66	2.77586E-54
AATF	EIF2B1	protein_coding	0.698	3.30089E-63
AATF	TBRG4	protein_coding	0.64	3.59886E-50
AATF	TLK2	protein_coding	0.654	4.67354E-53
AATF	TRIM28	protein_coding	0.674	2.2343E-57
AATF	USP21	protein_coding	0.606	8.59008E-44
AATF	CCDC34	protein_coding	0.632	1.00113E-48
AATF	C17orf53	protein_coding	0.643	8.25385E-51
AATF	SNX11	protein_coding	0.698	2.83938E-63

AATF	ANKS3	protein_coding	0.628	7.77535E-48
AATF	DBNL	protein_coding	0.64	3.87433E-50
AATF	NSMCE2	protein_coding	0.666	1.16284E-55
AATF	DDX42	protein_coding	0.609	2.36007E-44
AATF	UCKL1	protein_coding	0.655	3.26501E-53
AATF	GPRIN1	protein_coding	0.602	4.4641E-43
AATF	SF3B6	protein_coding	0.648	8.19736E-52
AATF	BZW2	protein_coding	0.618	5.34231E-46
AATF	AIMP1	protein_coding	0.616	1.43754E-45
AATF	HMGXB3	protein_coding	0.607	4.56074E-44
AATF	CSTF2	protein_coding	0.667	6.00937E-56
AATF	HINFP	protein_coding	0.616	1.1876E-45
AATF	UBE2C	protein_coding	0.641	1.8085E-50
AATF	MLST8	protein_coding	0.625	2.10641E-47
AATF	HMGA1	protein_coding	0.72	5.591E-69
AATF	SCNM1	protein_coding	0.734	5.17143E-73
AATF	LCMT1	protein_coding	0.696	1.09313E-62
AATF	MFSD5	protein_coding	0.706	2.40554E-65
AATF	ZSWIM9	protein_coding	0.642	1.18709E-50
AATF	UNC45A	protein_coding	0.65	2.70468E-52
AATF	PRCC	protein_coding	0.688	1.11522E-60
AATF	FEN1	protein_coding	0.638	8.6149E-50
AATF	DHX8	protein_coding	0.666	1.32643E-55
AATF	CCDC86	protein_coding	0.654	3.60346E-53
AATF	ELOVL1	protein_coding	0.654	4.51079E-53
AATF	DHX57	protein_coding	0.698	2.86258E-63
AATF	CRTC2	protein_coding	0.679	1.04917E-58
AATF	EME1	protein_coding	0.643	8.48601E-51
AATF	POLD1	protein_coding	0.679	1.01458E-58
AATF	PSMB5	protein_coding	0.625	2.99999E-47
AATF	CEP89	protein_coding	0.628	5.86263E-48
AATF	PCNA	protein_coding	0.652	1.26907E-52
AATF	TEPSIN	protein_coding	0.652	1.04369E-52
AATF	G6PD	protein_coding	0.677	4.67641E-58
AATF	DTX2	protein_coding	0.606	7.30612E-44
AATF	CIZ1	protein_coding	0.685	6.26186E-60
AATF	CSNK2B	protein_coding	0.608	3.74418E-44
AATF	EIF3M	protein_coding	0.694	2.74834E-62
AATF	DCAF13	protein_coding	0.601	5.26972E-43
AATF	TRMT112	protein_coding	0.655	2.92897E-53
AATF	NCAPH2	protein_coding	0.678	2.79497E-58
AATF	OTUB1	protein_coding	0.622	1.08262E-46
AATF	MRPL9	protein_coding	0.702	3.29449E-64
AATF	GSK3A	protein_coding	0.618	6.0935E-46

AATF	PPHLN1	protein_coding	0.616	9.68417E-46
AATF	DDX23	protein_coding	0.634	3.97658E-49
AATF	DEDD	protein_coding	0.606	8.13519E-44
AATF	SMYD5	protein_coding	0.723	6.96246E-70
AATF	SUPT6H	protein_coding	0.68	8.54566E-59
AATF	ILF3	protein_coding	0.643	8.68259E-51
AATF	DOHH	protein_coding	0.606	6.01647E-44
AATF	WDR62	protein_coding	0.604	1.62814E-43
AATF	AP4M1	protein_coding	0.656	2.00507E-53
AATF	AP5Z1	protein_coding	0.722	1.72228E-69
AATF	RNF25	protein_coding	0.653	6.17036E-53
AATF	UTP14A	protein_coding	0.709	5.50342E-66
AATF	PSMD8	protein_coding	0.622	1.05178E-46
AATF	LEMD2	protein_coding	0.656	1.80718E-53
AATF	RNF8	protein_coding	0.62	2.50538E-46
AATF	DSN1	protein_coding	0.623	5.2745E-47
AATF	C18orf21	protein_coding	0.684	6.83695E-60
AATF	SFR1	protein_coding	0.606	6.95351E-44
AATF	DDX49	protein_coding	0.664	2.43808E-55
AATF	PRIM1	protein_coding	0.646	2.04308E-51
AATF	RPL23	protein_coding	0.696	8.78007E-63
AATF	U2AF2	protein_coding	0.674	2.16236E-57
AATF	HCFC1	protein_coding	0.611	7.92128E-45
AATF	NOL7	protein_coding	0.711	1.23627E-66
AATF	MYO19	protein_coding	0.668	3.44978E-56
AATF	TCF3	protein_coding	0.635	2.52696E-49
AATF	TMEM115	protein_coding	0.654	4.70032E-53
AATF	BUB1	protein_coding	0.626	1.55633E-47
AATF	DCTPP1	protein_coding	0.67	1.61354E-56
AATF	ISY1	protein_coding	0.625	2.31013E-47
AATF	RARS	protein_coding	0.686	3.37578E-60
AATF	ABCF2	protein_coding	0.686	3.34459E-60
AATF	ZMYND19	protein_coding	0.629	3.77459E-48
AATF	PSMD4	protein_coding	0.703	1.4237E-64
AATF	UBE2M	protein_coding	0.662	9.83773E-55
AATF	DRG1	protein_coding	0.696	9.5259E-63
AATF	CUTA	protein_coding	0.608	3.59561E-44
AATF	DPY30	protein_coding	0.641	1.76033E-50
AATF	DDX56	protein_coding	0.686	2.71816E-60
AATF	SYNGR2	protein_coding	0.659	3.67438E-54
AATF	C15orf40	protein_coding	0.625	3.01357E-47
AATF	NAGPA	protein_coding	0.623	6.71212E-47
AATF	EIF3B	protein_coding	0.742	1.99506E-75
AATF	DDX54	protein_coding	0.696	1.28363E-62

AATF	PA2G4	protein_coding	0.73	7.46684E-72
AATF	PGS1	protein_coding	0.671	8.38274E-57
AATF	UTP6	protein_coding	0.833	1.3358E-110
AATF	MAFG	protein_coding	0.638	6.88544E-50
AATF	DYNLRB1	protein_coding	0.665	2.24537E-55
AATF	DDX1	protein_coding	0.614	3.23444E-45
AATF	SDF2	protein_coding	0.706	3.63597E-65
AATF	SCRIB	protein_coding	0.604	1.49523E-43
AATF	FTSJ1	protein_coding	0.675	1.03623E-57
AATF	EIF2B5	protein_coding	0.755	2.18921E-79
AATF	PPP1R35	protein_coding	0.622	8.66013E-47
AATF	SNRPA1	protein_coding	0.605	1.21887E-43
AATF	MED22	protein_coding	0.618	5.98989E-46
AATF	MCM4	protein_coding	0.603	2.17122E-43
AATF	SAMD1	protein_coding	0.608	3.08888E-44
AATF	SUMO2	protein_coding	0.688	1.02278E-60
AATF	SAFB	protein_coding	0.621	1.31309E-46
AATF	EIF2D	protein_coding	0.657	8.46117E-54
AATF	CDK7	protein_coding	0.634	3.99957E-49
AATF	ERGIC3	protein_coding	0.606	8.78073E-44
AATF	COG2	protein_coding	0.629	3.99597E-48
AATF	KHDRBS1	protein_coding	0.67	1.70107E-56
AATF	STX6	protein_coding	0.609	2.3016E-44
AATF	XAB2	protein_coding	0.618	5.40942E-46
AATF	NUSAP1	protein_coding	0.616	1.23233E-45
AATF	DIS3L2	protein_coding	0.604	1.71914E-43
AATF	CEP250	protein_coding	0.662	8.71513E-55
AATF	CCT3	protein_coding	0.757	4.00813E-80
AATF	ARPC3	protein_coding	0.613	4.19923E-45
AATF	IK	protein_coding	0.748	2.97724E-77
AATF	RBM28	protein_coding	0.623	6.86479E-47
AATF	AARSD1	protein_coding	0.674	1.94694E-57
AATF	CACYBP	protein_coding	0.703	2.09858E-64
AATF	BUB3	protein_coding	0.604	1.65607E-43
AATF	TRIM11	protein_coding	0.7	1.06395E-63
AATF	CD2BP2	protein_coding	0.689	4.44941E-61
AATF	BUD31	protein_coding	0.663	5.90158E-55
AATF	RBMX2	protein_coding	0.651	1.86917E-52
AATF	CDPF1	protein_coding	0.61	1.23547E-44
AATF	DDX27	protein_coding	0.763	6.78931E-82
AATF	NDUFAF2	protein_coding	0.628	8.11934E-48
AATF	ESS2	protein_coding	0.657	7.97339E-54
AATF	SLC35C2	protein_coding	0.65	2.8371E-52
AATF	NUP37	protein_coding	0.729	2.45621E-71

AATF	GPATCH3	protein_coding	0.653	8.33904E-53
AATF	ZMAT2	protein_coding	0.602	3.73872E-43
AATF	ZNF205	protein_coding	0.651	1.58477E-52
AATF	POLR1C	protein_coding	0.628	8.31868E-48
AATF	STMN1	protein_coding	0.681	3.86377E-59
AATF	WASHC2A	protein_coding	0.633	6.06224E-49
AATF	SUV39H1	protein_coding	0.633	7.92198E-49
AATF	TSN	protein_coding	0.601	5.87842E-43
AATF	PELP1	protein_coding	0.602	4.26385E-43
AATF	KIF11	protein_coding	0.606	6.11756E-44
AATF	MIIP	protein_coding	0.642	1.22484E-50
AATF	ZNF174	protein_coding	0.683	1.66965E-59
AATF	ANAPC5	protein_coding	0.607	5.96407E-44
AATF	CCDC124	protein_coding	0.631	1.87713E-48
AATF	COMMD5	protein_coding	0.605	1.13553E-43
AATF	MEA1	protein_coding	0.678	2.27263E-58
AATF	NOL11	protein_coding	0.682	2.06649E-59
AATF	VAT1	protein_coding	0.606	7.34153E-44
AATF	ANKRD27	protein_coding	0.633	7.60048E-49
AATF	FLAD1	protein_coding	0.671	8.48511E-57
AATF	RUSC1	protein_coding	0.627	1.00765E-47
AATF	USP39	protein_coding	0.642	1.234E-50
AATF	SCAF1	protein_coding	0.653	5.55379E-53
AATF	MRTO4	protein_coding	0.661	1.23887E-54
AATF	UBL7	protein_coding	0.643	8.48821E-51
AATF	TLCD1	protein_coding	0.619	2.82627E-46
AATF	CDCA5	protein_coding	0.663	4.98388E-55
AATF	OLA1	protein_coding	0.625	2.90091E-47
AATF	CDC20	protein_coding	0.677	4.66274E-58
AATF	HNRNPC	protein_coding	0.63	2.3537E-48
AATF	WDR75	protein_coding	0.62	2.1965E-46
AATF	CASC3	protein_coding	0.731	3.46214E-72
AATF	KIF20A	protein_coding	0.625	2.28545E-47
AATF	RUNDC1	protein_coding	0.639	4.23913E-50
AATF	PHF5A	protein_coding	0.607	4.0206E-44
AATF	KPNB1	protein_coding	0.673	3.5369E-57
AATF	NKIRAS2	protein_coding	0.75	1.00036E-77
AATF	MAPKAPK5	protein_coding	0.617	6.94155E-46
AATF	CCDC22	protein_coding	0.659	3.03336E-54
AATF	CNOT11	protein_coding	0.606	8.30927E-44
AATF	DDX39A	protein_coding	0.678	2.81077E-58
AATF	WDR46	protein_coding	0.744	4.88853E-76
AATF	POLG2	protein_coding	0.658	6.15563E-54
AATF	RRM2	protein_coding	0.641	2.23261E-50

AATF	NR2C2AP	protein_coding	0.748	3.11169E-77
AATF	RBBP7	protein_coding	0.614	2.81146E-45
AATF	DHX34	protein_coding	0.675	1.25686E-57
AATF	RPL27	protein_coding	0.679	1.39141E-58
AATF	MYBL2	protein_coding	0.662	7.7663E-55
AATF	SLC26A11	protein_coding	0.648	6.10094E-52
AATF	MED19	protein_coding	0.631	1.61048E-48
AATF	TCF19	protein_coding	0.613	3.84585E-45
AATF	BIRC5	protein_coding	0.681	5.75985E-59
AATF	RPP21	protein_coding	0.689	5.00209E-61
AATF	KIF4A	protein_coding	0.647	1.06702E-51
AATF	SUPT4H1	protein_coding	0.726	1.62615E-70
AATF	NLE1	protein_coding	0.768	1.05473E-83
AATF	GTPBP4	protein_coding	0.626	1.32621E-47
AATF	MRPL58	protein_coding	0.618	4.48034E-46
AATF	RALY	protein_coding	0.74	8.96667E-75
AATF	RAB13	protein_coding	0.61	1.34242E-44
AATF	PARP1	protein_coding	0.621	1.35609E-46
AATF	EWSR1	protein_coding	0.659	4.21744E-54
AATF	AHSA1	protein_coding	0.612	5.2467E-45
AATF	RAD51C	protein_coding	0.652	1.3887E-52
AATF	ATRAID	protein_coding	0.626	1.7671E-47
AATF	CFL1	protein_coding	0.638	9.22982E-50
AATF	CDK5RAP1	protein_coding	0.695	1.86955E-62
AATF	GNL2	protein_coding	0.628	7.20586E-48
AATF	WRAP73	protein_coding	0.635	3.03673E-49
AATF	SNRPB	protein_coding	0.766	4.49869E-83
AATF	BRIX1	protein_coding	0.678	2.23935E-58
AATF	RBM45	protein_coding	0.669	2.24638E-56
AATF	NXT1	protein_coding	0.612	6.44649E-45
AATF	EXOC3	protein_coding	0.681	3.98682E-59
AATF	UBE2S	protein_coding	0.667	8.28671E-56
AATF	MAF1	protein_coding	0.609	2.20025E-44
AATF	PIGU	protein_coding	0.678	1.92726E-58
AATF	PDCD6	protein_coding	0.64	3.17192E-50
AATF	RTF2	protein_coding	0.682	2.86304E-59
AATF	SF3A2	protein_coding	0.673	2.88785E-57
AATF	SNRPD2	protein_coding	0.647	1.31066E-51
AATF	MRPL48	protein_coding	0.603	2.21311E-43
AATF	RRP36	protein_coding	0.699	1.94616E-63
AATF	RAB5C	protein_coding	0.614	3.19261E-45
AATF	DTL	protein_coding	0.606	8.55289E-44
AATF	DRAP1	protein_coding	0.604	1.55178E-43
AATF	NCAPH	protein_coding	0.656	1.34833E-53

AATF	SUPT5H	protein_coding	0.661	1.43512E-54
AATF	TAF15	protein_coding	0.754	3.40874E-79
AATF	NME1	protein_coding	0.686	3.62602E-60
AATF	CDK4	protein_coding	0.604	1.79831E-43
AATF	MKS1	protein_coding	0.64	3.48169E-50
AATF	TFAP4	protein_coding	0.644	5.45985E-51
AATF	ATP6AP1	protein_coding	0.662	8.81596E-55
AATF	METTL2A	protein_coding	0.673	3.94473E-57
AATF	CDKN3	protein_coding	0.633	6.97545E-49
AATF	EIF2S2	protein_coding	0.658	6.60464E-54
AATF	PFDN6	protein_coding	0.674	1.98851E-57
AATF	UBE2O	protein_coding	0.699	1.87651E-63
AATF	POP5	protein_coding	0.62	2.68241E-46
AATF	TRIP4	protein_coding	0.664	2.73662E-55
AATF	APEX2	protein_coding	0.634	5.084E-49
AATF	BRMS1	protein_coding	0.669	2.01254E-56
AATF	RFT1	protein_coding	0.616	1.20101E-45
AATF	CMSS1	protein_coding	0.705	6.66615E-65
AATF	SRP68	protein_coding	0.685	6.16633E-60
AATF	LIN37	protein_coding	0.647	1.01162E-51
AATF	MUTYH	protein_coding	0.644	5.83296E-51
AATF	HDAC5	protein_coding	0.638	9.34047E-50
AATF	GNL1	protein_coding	0.678	2.29628E-58
AATF	TELO2	protein_coding	0.635	2.83236E-49
AATF	HDAC11	protein_coding	0.637	1.33046E-49
AATF	CC2D1B	protein_coding	0.67	1.55674E-56
AATF	COIL	protein_coding	0.643	9.53223E-51
AATF	UNK	protein_coding	0.642	1.23037E-50
AATF	KIF2C	protein_coding	0.673	2.97003E-57
AATF	RNASEH2A	protein_coding	0.707	1.67087E-65
AATF	CSNK1D	protein_coding	0.719	1.14897E-68
AATF	FAM220A	protein_coding	0.69	3.25071E-61
AATF	C8orf59	protein_coding	0.606	6.75963E-44
AATF	SIRT6	protein_coding	0.655	2.26047E-53
AATF	XPO6	protein_coding	0.624	3.24E-47
AATF	GPBP1	protein_coding	0.623	5.82133E-47
AATF	IDH3B	protein_coding	0.604	1.7363E-43
AATF	MRPL33	protein_coding	0.607	5.02835E-44
AATF	CDKN2AIPNL	protein_coding	0.682	2.35392E-59
AATF	CBX8	protein_coding	0.604	1.40382E-43
AATF	PYCR2	protein_coding	0.671	6.99258E-57
AATF	DHX37	protein_coding	0.686	2.56204E-60
AATF	HSP90AB1	protein_coding	0.675	8.49185E-58
AATF	YKT6	protein_coding	0.632	1.08348E-48

AATF	STK11IP	protein_coding	0.616	1.26359E-45
AATF	SNU13	protein_coding	0.634	4.02993E-49
AATF	PCLAF	protein_coding	0.648	6.33627E-52
AATF	DPM2	protein_coding	0.637	1.50934E-49
AATF	TMEM14C	protein_coding	0.618	4.80251E-46
AATF	FKBP1A	protein_coding	0.609	1.9541E-44
AATF	COPRS	protein_coding	0.641	1.83342E-50
AATF	THAP11	protein_coding	0.64	3.12565E-50
AATF	DDA1	protein_coding	0.645	3.63278E-51
AATF	URM1	protein_coding	0.629	3.62151E-48
AATF	SPDL1	protein_coding	0.632	1.23669E-48
AATF	ILF2	protein_coding	0.738	3.44054E-74
AATF	COMMD4	protein_coding	0.624	4.41863E-47
AATF	SPC24	protein_coding	0.625	2.05038E-47
AATF	PSMC3IP	protein_coding	0.615	1.65326E-45
AATF	NEU1	protein_coding	0.657	9.35723E-54
AATF	FAF1	protein_coding	0.656	1.35301E-53
AATF	UBTF	protein_coding	0.673	2.90802E-57
AATF	HNRNPM	protein_coding	0.622	8.50817E-47
AATF	MRPL10	protein_coding	0.76	4.83387E-81
AATF	ZNF692	protein_coding	0.629	4.5863E-48
AATF	ABCF3	protein_coding	0.629	4.47366E-48
AATF	WIPF2	protein_coding	0.631	1.88443E-48
AATF	TMEM120B	protein_coding	0.627	9.5139E-48
AATF	BMS1	protein_coding	0.645	3.43435E-51
AATF	RAD1	protein_coding	0.619	2.85596E-46
AATF	CWC27	protein_coding	0.737	1.06421E-73
AATF	CCT7	protein_coding	0.759	8.35848E-81
AATF	MRPS7	protein_coding	0.654	3.56541E-53
AATF	EIF1AD	protein_coding	0.639	5.08378E-50
AATF	ITGB1BP1	protein_coding	0.643	8.99834E-51
AATF	CANT1	protein_coding	0.699	1.58966E-63
AATF	THOC6	protein_coding	0.656	1.45145E-53
AATF	IQCC	protein_coding	0.628	7.87422E-48
AATF	ZNF207	protein_coding	0.679	1.54054E-58
AATF	METTL1	protein_coding	0.662	9.58156E-55
AATF	CCDC43	protein_coding	0.681	4.33281E-59
AATF	SKIV2L	protein_coding	0.65	3.66543E-52
AATF	EHMT2	protein_coding	0.758	3.35998E-80
AATF	VPS25	protein_coding	0.731	5.30288E-72
AATF	TCOF1	protein_coding	0.788	9.49048E-91
AATF	BOP1	protein_coding	0.611	8.29691E-45
AATF	KRI1	protein_coding	0.672	5.49221E-57
AATF	NOP56	protein_coding	0.707	1.3152E-65

AATF	E2F1	protein_coding	0.612	7.31636E-45
AATF	COPS6	protein_coding	0.695	2.37412E-62
AATF	GPS1	protein_coding	0.675	1.36781E-57
AATF	RPIA	protein_coding	0.644	4.70994E-51
AATF	HARS2	protein_coding	0.683	1.84049E-59
AATF	GPKOW	protein_coding	0.682	3.1925E-59
AATF	TTC9C	protein_coding	0.607	5.56582E-44
AATF	C17orf80	protein_coding	0.602	4.33806E-43
AATF	ACD	protein_coding	0.729	1.33347E-71
AATF	TPRKB	protein_coding	0.691	1.50361E-61
AATF	KRTCAP2	protein_coding	0.631	1.7343E-48
AATF	PFDN1	protein_coding	0.634	4.71443E-49
AATF	ABCF1	protein_coding	0.703	1.50892E-64
AATF	NABP2	protein_coding	0.743	1.17437E-75
AATF	SSB	protein_coding	0.621	1.42665E-46
AATF	TIGD5	protein_coding	0.617	8.50337E-46
AATF	REXO4	protein_coding	0.677	3.80013E-58
AATF	WDR74	protein_coding	0.616	1.45561E-45
AATF	SMG9	protein_coding	0.617	6.48266E-46
AATF	HNRNPA1	protein_coding	0.643	6.6427E-51
AATF	MAD2L1BP	protein_coding	0.607	4.01465E-44
AATF	RAE1	protein_coding	0.69	2.93372E-61
AATF	MED24	protein_coding	0.724	3.78029E-70
AATF	RBM17	protein_coding	0.602	4.46182E-43
AATF	METTL18	protein_coding	0.62	2.17951E-46
AATF	TMUB2	protein_coding	0.701	6.45805E-64
AATF	CKS2	protein_coding	0.616	1.28771E-45
AATF	ZNF622	protein_coding	0.607	5.64924E-44
AATF	CHAF1A	protein_coding	0.636	2.06571E-49
AATF	HAUS1	protein_coding	0.65	2.85667E-52
AATF	NMT1	protein_coding	0.626	1.56318E-47
AATF	TMEM201	protein_coding	0.619	3.47433E-46
AATF	SSR2	protein_coding	0.603	2.98295E-43
AATF	C12orf10	protein_coding	0.69	3.12532E-61
AATF	LSM4	protein_coding	0.625	2.27938E-47
AATF	RPL38	protein_coding	0.623	5.00993E-47
AATF	NUF2	protein_coding	0.627	9.74304E-48
AATF	GM2A	protein_coding	0.604	1.83215E-43
AATF	MANBAL	protein_coding	0.68	8.54084E-59
AATF	NOC4L	protein_coding	0.648	7.93031E-52
AATF	PTTG1	protein_coding	0.698	2.90302E-63
AATF	AGBL5	protein_coding	0.662	7.14007E-55
AATF	PYM1	protein_coding	0.641	2.26288E-50
AATF	AURKB	protein_coding	0.659	2.90522E-54

AATF	ATP6V1E1	protein_coding	0.673	3.82633E-57
AATF	HDGFL2	protein_coding	0.666	1.19677E-55
AATF	LSM2	protein_coding	0.724	4.39188E-70
AATF	RBM15B	protein_coding	0.606	7.02801E-44
AATF	XYLT2	protein_coding	0.66	2.06865E-54
AATF	SNRPD1	protein_coding	0.677	4.32086E-58
AATF	LAS1L	protein_coding	0.694	2.74961E-62
AATF	ZNF346	protein_coding	0.63	3.15514E-48
AATF	ALG3	protein_coding	0.632	1.21506E-48
AATF	MTFR2	protein_coding	0.623	5.26291E-47
AATF	RBM14	protein_coding	0.649	4.77484E-52
AATF	ANKZF1	protein_coding	0.685	4.23282E-60
AATF	SAFB2	protein_coding	0.65	3.60085E-52
AATF	STX4	protein_coding	0.689	6.40058E-61
AATF	HGS	protein_coding	0.607	4.07683E-44
AATF	CYBC1	protein_coding	0.631	2.14423E-48
AATF	PDCD2L	protein_coding	0.621	1.37224E-46
AATF	PWP1	protein_coding	0.711	2.10599E-66
AATF	DPF2	protein_coding	0.617	8.52691E-46
AATF	DTNB	protein_coding	0.635	3.68067E-49
AATF	CEP131	protein_coding	0.765	8.12774E-83
AATF	EXOSC1	protein_coding	0.668	4.37404E-56
AATF	DNTTIP2	protein_coding	0.65	2.65483E-52
AATF	TPM3	protein_coding	0.681	3.95089E-59
AATF	CKS1B	protein_coding	0.711	1.3775E-66
AATF	PHF14	protein_coding	0.617	7.2695E-46
AATF	GPATCH4	protein_coding	0.666	1.21488E-55
AATF	EXO1	protein_coding	0.65	2.32126E-52
AATF	RPUSD1	protein_coding	0.66	2.37026E-54
AATF	NDC80	protein_coding	0.627	1.02552E-47
AATF	THOC7	protein_coding	0.625	2.94332E-47
AATF	RBM22	protein_coding	0.685	4.77391E-60
AATF	HAUS5	protein_coding	0.668	4.05201E-56
AATF	KXD1	protein_coding	0.694	3.31925E-62
AATF	RAB24	protein_coding	0.637	1.05008E-49
AATF	NAT10	protein_coding	0.652	1.11234E-52
AATF	MED10	protein_coding	0.622	9.85997E-47
AATF	GTPBP3	protein_coding	0.626	1.48304E-47
AATF	ZWINT	protein_coding	0.641	2.28045E-50
AATF	PPIA	protein_coding	0.68	6.50298E-59
AATF	RACGAP1	protein_coding	0.675	1.33427E-57
AATF	TMEM206	protein_coding	0.67	1.51568E-56
AATF	PDZD11	protein_coding	0.663	4.34254E-55
AATF	MCM6	protein_coding	0.657	9.47606E-54

AATF	PES1	protein_coding	0.69	2.87289E-61
AATF	EIF4A3	protein_coding	0.745	4.35921E-76
AATF	SLC4A1AP	protein_coding	0.639	4.37426E-50
AATF	PRPF31	protein_coding	0.609	2.62294E-44
AATF	PSMD2	protein_coding	0.636	2.30089E-49
AATF	PIP4K2B	protein_coding	0.66	2.36052E-54
AATF	ARPC5	protein_coding	0.616	1.10826E-45
AATF	NELFE	protein_coding	0.756	8.33869E-80
AATF	AAAS	protein_coding	0.702	3.05736E-64
AATF	GDI1	protein_coding	0.611	8.44897E-45
AATF	ANP32A	protein_coding	0.605	1.07517E-43
AATF	MRGBP	protein_coding	0.678	1.90009E-58
AATF	TSG101	protein_coding	0.627	1.22144E-47
AATF	SMG5	protein_coding	0.665	1.44254E-55
AATF	GMPPA	protein_coding	0.609	2.15528E-44
AATF	PRELID1	protein_coding	0.608	2.84836E-44
AATF	TUBB	protein_coding	0.682	2.59491E-59
AATF	NVL	protein_coding	0.663	4.88478E-55
AATF	FANCG	protein_coding	0.672	5.96296E-57
AATF	USF1	protein_coding	0.627	9.67654E-48
AATF	MTA3	protein_coding	0.638	8.88495E-50
AATF	INTS8	protein_coding	0.632	1.35768E-48
AATF	TOP2A	protein_coding	0.642	1.04725E-50
AATF	NIFK	protein_coding	0.609	1.82363E-44
AATF	NARF	protein_coding	0.656	1.3351E-53
AATF	COPS7B	protein_coding	0.624	3.33943E-47
AATF	GGA3	protein_coding	0.655	2.26175E-53
AATF	LRRC59	protein_coding	0.673	3.03009E-57
AATF	TIPIN	protein_coding	0.629	3.60054E-48
AATF	UTP18	protein_coding	0.801	3.4319E-96
AATF	RPS7	protein_coding	0.604	1.59347E-43
AATF	CDCA8	protein_coding	0.634	5.00084E-49
AATF	VPS33A	protein_coding	0.658	7.6027E-54
AATF	CCNF	protein_coding	0.621	1.52234E-46
AATF	MAGOH	protein_coding	0.669	2.11208E-56
AATF	EEF1E1	protein_coding	0.603	2.02554E-43
AATF	SNRPC	protein_coding	0.757	6.59512E-80
AATF	AP2M1	protein_coding	0.621	1.49432E-46
AATF	SLC39A1	protein_coding	0.628	6.43863E-48
AATF	MED15	protein_coding	0.601	6.61866E-43
AATF	NEK2	protein_coding	0.64	3.30802E-50
AATF	FKBPL	protein_coding	0.656	1.4859E-53
AATF	ERAL1	protein_coding	0.763	5.36527E-82
AATF	VPS45	protein_coding	0.642	1.22057E-50

AATF	POC5	protein_coding	0.63	2.31975E-48
AATF	CDC123	protein_coding	0.664	3.47116E-55
AATF	ZMAT5	protein_coding	0.634	3.87474E-49
AATF	TRMU	protein_coding	0.611	8.59087E-45
AATF	SNAPIN	protein_coding	0.676	6.97697E-58
AATF	DUSP12	protein_coding	0.672	6.17468E-57
AATF	MCM2	protein_coding	0.652	1.00681E-52
AATF	CCNB2	protein_coding	0.645	2.8923E-51
AATF	PQBP1	protein_coding	0.655	2.39453E-53
AATF	RECQL4	protein_coding	0.64	2.84111E-50
AATF	DTYMK	protein_coding	0.697	6.92324E-63
AATF	MCM7	protein_coding	0.697	6.73404E-63
AATF	FTSJ3	protein_coding	0.767	1.999E-83
AATF	LMNB2	protein_coding	0.638	7.82242E-50
AATF	MRPL45	protein_coding	0.713	3.86555E-67
AATF	NUP155	protein_coding	0.614	2.57518E-45
AATF	DDX52	protein_coding	0.708	9.23087E-66
AATF	GTF2F1	protein_coding	0.628	6.24812E-48
AATF	FARSB	protein_coding	0.688	9.0013E-61
AATF	PPIH	protein_coding	0.657	1.04098E-53
AATF	CHTOP	protein_coding	0.662	6.54004E-55
AATF	RFXANK	protein_coding	0.709	4.94191E-66
AATF	HJURP	protein_coding	0.618	5.2044E-46
AATF	NRBP1	protein_coding	0.686	2.6417E-60
AATF	BANF1	protein_coding	0.63	2.305E-48
AATF	SCAMP3	protein_coding	0.746	1.9737E-76
AATF	UBE2I	protein_coding	0.676	6.22728E-58
AATF	CRNKL1	protein_coding	0.652	1.27047E-52
AATF	UIMC1	protein_coding	0.643	7.463E-51
AATF	NCAPG	protein_coding	0.64	2.96117E-50
AATF	MCM3	protein_coding	0.674	1.99874E-57
AATF	MED8	protein_coding	0.643	7.28874E-51
AATF	LMNA	protein_coding	0.644	6.16186E-51
AATF	PFDN4	protein_coding	0.627	1.19569E-47
AATF	ATP6V1F	protein_coding	0.632	1.34716E-48
AATF	SART1	protein_coding	0.719	8.31862E-69
AATF	ADSL	protein_coding	0.669	2.16154E-56
AATF	EXOSC5	protein_coding	0.634	4.20027E-49
AATF	MAN1B1	protein_coding	0.614	2.73875E-45
AATF	TRNAU1AP	protein_coding	0.656	1.45125E-53
AATF	MRM1	protein_coding	0.652	9.63504E-53
AATF	ITPA	protein_coding	0.637	1.35913E-49
AATF	CDK5	protein_coding	0.613	4.2136E-45
AATF	PSMD13	protein_coding	0.606	6.19897E-44

AATF	IRF3	protein_coding	0.612	7.57913E-45
AATF	PRKAG1	protein_coding	0.651	2.17105E-52
AATF	PRC1	protein_coding	0.631	1.80966E-48
AATF	DCTN1	protein_coding	0.627	1.17098E-47
AATF	HNRNPL	protein_coding	0.667	6.97135E-56
AATF	HNRNPA3	protein_coding	0.641	2.30659E-50
AATF	BTF3	protein_coding	0.627	1.23882E-47
AATF	UBE2T	protein_coding	0.722	2.2814E-69
AATF	ASF1B	protein_coding	0.644	5.32706E-51
AATF	FXR1	protein_coding	0.614	3.20527E-45
AATF	EIF3G	protein_coding	0.608	2.76872E-44
AATF	DROSHA	protein_coding	0.629	4.99921E-48
AATF	FOXK2	protein_coding	0.628	7.37937E-48
AATF	CENPW	protein_coding	0.653	6.86747E-53
AATF	TPD52L2	protein_coding	0.7	1.18378E-63
AATF	MSTO1	protein_coding	0.637	1.34103E-49
AATF	GLMN	protein_coding	0.602	3.51706E-43
AATF	CCDC77	protein_coding	0.633	7.23882E-49
AATF	PPP1CC	protein_coding	0.656	1.30427E-53
AATF	ITGB3BP	protein_coding	0.632	1.38169E-48
AATF	UCK2	protein_coding	0.687	2.20219E-60
AATF	NSFL1C	protein_coding	0.671	8.94858E-57
AATF	TMEM199	protein_coding	0.77	1.58058E-84
AATF	COLGALT1	protein_coding	0.618	5.64436E-46
AATF	FAM189B	protein_coding	0.691	1.58981E-61
AATF	KAT2A	protein_coding	0.712	1.09757E-66
AATF	GINS1	protein_coding	0.623	7.2227E-47
AATF	PRR3	protein_coding	0.631	1.6772E-48
AATF	H2AFX	protein_coding	0.642	1.43528E-50
AATF	BLOC1S3	protein_coding	0.655	3.26308E-53
AATF	PPOX	protein_coding	0.669	2.72147E-56
AATF	TIGD6	protein_coding	0.611	8.60229E-45
AATF	GTF3C2	protein_coding	0.631	1.6028E-48
AATF	INTS11	protein_coding	0.611	1.03315E-44
AATF	CXXC1	protein_coding	0.626	1.87748E-47
AATF	RPP38	protein_coding	0.678	1.95514E-58
AATF	ZBTB17	protein_coding	0.643	8.07145E-51
AATF	GMEB2	protein_coding	0.615	1.79011E-45
AATF	TAF11	protein_coding	0.629	4.24222E-48
AATF	LLPH	protein_coding	0.623	7.43397E-47
AATF	IGBP1	protein_coding	0.647	1.00406E-51
AATF	TRMT6	protein_coding	0.681	4.49441E-59
AATF	MFAP1	protein_coding	0.616	1.35589E-45
AATF	SF3B2	protein_coding	0.658	5.58191E-54

AATF	GTF3C5	protein_coding	0.637	1.52868E-49
AATF	PSMB3	protein_coding	0.664	2.65425E-55
AATF	CDK5RAP3	protein_coding	0.666	1.40032E-55
AATF	NONO	protein_coding	0.652	1.09593E-52
AATF	CDCA3	protein_coding	0.632	9.90194E-49
AATF	DNAJC8	protein_coding	0.627	9.35706E-48
AATF	DBF4B	protein_coding	0.651	1.65857E-52
AATF	INTS4	protein_coding	0.616	1.20504E-45
AATF	PRIM2	protein_coding	0.684	1.06712E-59
AATF	MCM5	protein_coding	0.645	2.43424E-51
AATF	SNAPC2	protein_coding	0.602	3.79767E-43
AATF	ACTR5	protein_coding	0.617	7.5145E-46
AATF	NAP1L4	protein_coding	0.61	1.5107E-44
AATF	SAC3D1	protein_coding	0.628	6.98572E-48
AATF	BAX	protein_coding	0.615	2.17569E-45
AATF	SKA1	protein_coding	0.634	4.35552E-49
AATF	NSF	protein_coding	0.723	8.26892E-70
AATF	EIF3H	protein_coding	0.607	5.60381E-44
AATF	YAE1	protein_coding	0.642	1.17713E-50
AATF	NFKBIL1	protein_coding	0.669	2.50072E-56
AATF	SRP14	protein_coding	0.601	5.41653E-43
AATF	RAC1	protein_coding	0.611	8.73754E-45
AATF	RBM8A	protein_coding	0.665	2.22855E-55
AATF	PRPF6	protein_coding	0.733	9.57751E-73
AATF	MORC2	protein_coding	0.605	1.1909E-43
AATF	SLC41A3	protein_coding	0.659	3.46523E-54
AATF	EDC3	protein_coding	0.703	1.52501E-64
AATF	SMARCA4	protein_coding	0.613	5.02176E-45
AATF	BYSL	protein_coding	0.661	1.27723E-54
AATF	NACA	protein_coding	0.678	1.74125E-58
AATF	LAMTOR5	protein_coding	0.647	1.12778E-51
AATF	CAD	protein_coding	0.663	4.51709E-55
AATF	NCAPD2	protein_coding	0.611	1.07662E-44
AATF	CHMP4B	protein_coding	0.639	5.98425E-50
AATF	VPS11	protein_coding	0.601	4.88022E-43
AATF	UFC1	protein_coding	0.659	2.98186E-54
AATF	PCGF1	protein_coding	0.677	3.09431E-58
AATF	NT5C3B	protein_coding	0.66	2.25379E-54
AATF	RECQL5	protein_coding	0.677	3.89448E-58
AATF	PSMA4	protein_coding	0.616	1.39535E-45
AATF	NUP107	protein_coding	0.604	1.75465E-43
AATF	TRMT61A	protein_coding	0.644	5.96625E-51
AATF	SLC50A1	protein_coding	0.615	2.12659E-45
AATF	SF3A3	protein_coding	0.668	3.96852E-56

AATF	SUGP1	protein_coding	0.707	2.00261E-65
AATF	AATF	protein_coding	1	0
AATF	PDCL3	protein_coding	0.68	6.92059E-59
AATF	UBAP2L	protein_coding	0.663	4.49427E-55
AATF	TBC1D31	protein_coding	0.61	1.40373E-44
AATF	NRF1	protein_coding	0.626	1.96316E-47
AATF	STK25	protein_coding	0.655	2.71758E-53
AATF	CDT1	protein_coding	0.688	7.52009E-61
AATF	TMEM79	protein_coding	0.624	3.62129E-47
AATF	ERI3	protein_coding	0.663	4.08228E-55
AATF	DNAJC7	protein_coding	0.813	3.1352E-101
AATF	GMPS	protein_coding	0.612	6.69406E-45
AATF	ACBD6	protein_coding	0.68	7.2939E-59
AATF	C20orf96	protein_coding	0.605	1.25642E-43
AATF	MAPK3	protein_coding	0.671	1.06025E-56
AATF	PKN1	protein_coding	0.663	5.93609E-55
AATF	CBX3	protein_coding	0.695	1.50904E-62
AATF	GPATCH1	protein_coding	0.609	1.86604E-44
AATF	CPSF4	protein_coding	0.662	1.02709E-54
AATF	ARFGAP1	protein_coding	0.651	1.48244E-52
AATF	UBE2Q1	protein_coding	0.663	4.08419E-55
AATF	FAM192A	protein_coding	0.623	6.18175E-47
AATF	RRP12	protein_coding	0.644	4.72155E-51
AATF	TOMM40	protein_coding	0.65	2.48169E-52
AATF	RPL23A	protein_coding	0.647	1.35085E-51
AATF	NT5C	protein_coding	0.667	6.27438E-56
AATF	CNP	protein_coding	0.602	4.17249E-43
AATF	CLIC1	protein_coding	0.667	6.22408E-56
AATF	PPM1G	protein_coding	0.761	2.44169E-81
AATF	SIRT7	protein_coding	0.737	7.36482E-74
AATF	CHCHD2	protein_coding	0.621	1.68096E-46
AATF	KCTD2	protein_coding	0.651	1.72486E-52
AATF	NRM	protein_coding	0.615	1.47146E-45
AATF	DDX41	protein_coding	0.688	9.94944E-61
AATF	KPNA2	protein_coding	0.714	2.56412E-67
AATF	EIF2B4	protein_coding	0.728	3.91185E-71
AATF	NGDN	protein_coding	0.665	1.76192E-55
AATF	NCL	protein_coding	0.613	3.40866E-45
AATF	MRPS23	protein_coding	0.779	1.93945E-87
AATF	CCT6A	protein_coding	0.713	4.24835E-67
AATF	CHEK1	protein_coding	0.62	2.56804E-46
AATF	FAM49B	protein_coding	0.636	1.67079E-49
AATF	PSMC4	protein_coding	0.649	4.56761E-52
AATF	ZNHIT3	protein_coding	0.829	2.3696E-108

AATF	CCHCR1	protein_coding	0.724	4.199E-70
AATF	TMEM147	protein_coding	0.61	1.56643E-44
AATF	TARDBP	protein_coding	0.63	3.00544E-48
AATF	CENPM	protein_coding	0.682	3.39074E-59
AATF	RPUSD3	protein_coding	0.617	9.31391E-46
AATF	TACC3	protein_coding	0.663	5.18397E-55
AATF	CCT2	protein_coding	0.716	9.44586E-68
AATF	TBC1D16	protein_coding	0.603	2.27189E-43
AATF	ATXN7L3	protein_coding	0.601	5.39457E-43
AATF	CLTA	protein_coding	0.706	3.44396E-65
AATF	SNF8	protein_coding	0.757	3.69456E-80
AATF	C14orf93	protein_coding	0.629	5.17102E-48
AATF	CLCN2	protein_coding	0.624	3.18732E-47
AATF	CPSF6	protein_coding	0.609	2.15709E-44
AATF	HSPA4	protein_coding	0.62	2.18858E-46
AATF	RUVBL2	protein_coding	0.647	9.59585E-52
AATF	TRIP13	protein_coding	0.672	5.46166E-57
AATF	KIFC1	protein_coding	0.689	6.85621E-61
AATF	NUP62	protein_coding	0.619	3.95195E-46
AATF	NEDD8	protein_coding	0.634	5.74229E-49
AATF	ASB6	protein_coding	0.614	3.33797E-45
AATF	RBM10	protein_coding	0.723	6.47886E-70
AATF	CBX1	protein_coding	0.678	2.58214E-58
AATF	RNPS1	protein_coding	0.692	1.27767E-61
AATF	NAP1L1	protein_coding	0.642	1.05286E-50
AATF	COA6	protein_coding	0.613	3.77831E-45
AATF	MRPL17	protein_coding	0.609	2.25091E-44
AATF	SNX8	protein_coding	0.643	7.15951E-51
AATF	BRAT1	protein_coding	0.677	4.24144E-58
AATF	PPME1	protein_coding	0.62	2.6648E-46
AATF	DYNC1LI1	protein_coding	0.612	5.97101E-45
AATF	SLC25A19	protein_coding	0.602	3.02309E-43
AATF	TXNDC9	protein_coding	0.605	1.10806E-43
AATF	FANCI	protein_coding	0.643	7.2347E-51
AATF	GNPDA1	protein_coding	0.605	1.23833E-43
AATF	AKAP8L	protein_coding	0.659	3.4222E-54
AATF	HDGF	protein_coding	0.605	1.04549E-43
AATF	CENPB	protein_coding	0.654	4.20969E-53
AATF	SURF6	protein_coding	0.613	3.81171E-45
AATF	DAD1	protein_coding	0.626	1.90037E-47
AATF	CCDC137	protein_coding	0.775	5.47312E-86
AATF	GGNBP2	protein_coding	0.648	9.03238E-52
AATF	SNRPE	protein_coding	0.707	1.41342E-65
AATF	NME6	protein_coding	0.691	2.12303E-61

AATF	PUF60	protein_coding	0.615	1.70129E-45
AATF	GBA	protein_coding	0.61	1.42721E-44
AATF	PRPF19	protein_coding	0.682	2.48851E-59
AATF	RAN	protein_coding	0.664	2.61213E-55
AATF	RABIF	protein_coding	0.617	8.29552E-46
AATF	NOP58	protein_coding	0.653	8.6283E-53
AATF	TOMM34	protein_coding	0.651	1.9573E-52
AATF	NPM1	protein_coding	0.666	9.49837E-56
AATF	ORC1	protein_coding	0.618	4.98994E-46
AATF	SF3B4	protein_coding	0.74	1.2018E-74
AATF	MTMR14	protein_coding	0.653	8.64756E-53
AATF	EPRS	protein_coding	0.611	8.82916E-45
AATF	POLR3C	protein_coding	0.726	1.21208E-70
AATF	CENPA	protein_coding	0.62	2.19389E-46
AATF	UTP11	protein_coding	0.607	4.89713E-44
AATF	HRAS	protein_coding	0.618	4.61848E-46
AATF	WDR4	protein_coding	0.682	2.00031E-59
AATF	VPS72	protein_coding	0.778	3.54106E-87
AATF	PTBP1	protein_coding	0.645	3.13635E-51
AATF	SNRPG	protein_coding	0.659	2.90484E-54
AATF	RUVBL1	protein_coding	0.741	7.22725E-75
AATF	RCCD1	protein_coding	0.618	5.75549E-46
AATF	B4GALT3	protein_coding	0.677	3.09408E-58
AATF	NAIF1	protein_coding	0.601	5.86126E-43
AATF	SPC25	protein_coding	0.675	1.3577E-57
AATF	PI4KB	protein_coding	0.623	6.32738E-47
AATF	DAP3	protein_coding	0.638	7.57402E-50
AATF	NAT9	protein_coding	0.736	2.25855E-73
AATF	RPL17	protein_coding	0.634	4.27141E-49
AATF	ATP5MC2	protein_coding	0.628	8.1135E-48
AATF	SUPT7L	protein_coding	0.616	1.26558E-45
AATF	POP4	protein_coding	0.632	9.29418E-49
AATF	KAT7	protein_coding	0.604	1.44695E-43
AATF	WDR53	protein_coding	0.651	2.22144E-52
AATF	BRAP	protein_coding	0.635	2.58624E-49
AATF	APEX1	protein_coding	0.621	1.40924E-46
AATF	MTCH1	protein_coding	0.647	1.00387E-51
AATF	POLR2G	protein_coding	0.69	2.87574E-61
AATF	STARD3	protein_coding	0.76	5.74171E-81
AATF	RHOA	protein_coding	0.606	8.25162E-44
AATF	ACTL6A	protein_coding	0.604	1.6829E-43
AATF	RHNO1	protein_coding	0.604	1.42243E-43
AATF	SPOP	protein_coding	0.601	4.63046E-43
AATF	XPO5	protein_coding	0.671	7.94296E-57

AATF	PIGT	protein_coding	0.639	5.53172E-50
AATF	COASY	protein_coding	0.684	8.34162E-60
AATF	TRAF2	protein_coding	0.664	3.93953E-55
AATF	DTNBP1	protein_coding	0.602	3.34717E-43
AATF	CCT5	protein_coding	0.689	4.99425E-61
AATF	MTX1	protein_coding	0.694	3.81506E-62
AATF	TUBD1	protein_coding	0.628	7.93321E-48
AATF	SFPQ	protein_coding	0.612	7.6978E-45
AATF	PSMD11	protein_coding	0.762	1.28784E-81
AATF	ALYREF	protein_coding	0.702	3.88525E-64
AATF	DENR	protein_coding	0.603	2.03897E-43
AATF	MSH2	protein_coding	0.633	8.05025E-49
AATF	C14orf119	protein_coding	0.629	3.85153E-48
AATF	AKIP1	protein_coding	0.671	1.07765E-56
AATF	BAG6	protein_coding	0.663	4.67339E-55
AATF	DGKZ	protein_coding	0.601	6.05484E-43
AATF	PSMC5	protein_coding	0.671	1.0148E-56
AATF	LRRC14	protein_coding	0.621	1.28938E-46
AATF	PDRG1	protein_coding	0.693	5.7042E-62
AATF	NUTF2	protein_coding	0.612	6.42556E-45
AATF	TMEM101	protein_coding	0.755	1.93192E-79
AATF	POLA2	protein_coding	0.608	2.90803E-44
AATF	GIT1	protein_coding	0.683	1.73838E-59
AATF	MAP1S	protein_coding	0.618	5.63773E-46
AATF	RBM19	protein_coding	0.65	2.55782E-52
AATF	ZNF526	protein_coding	0.658	5.95362E-54
AATF	NOL10	protein_coding	0.685	5.24691E-60
AATF	SNRPD3	protein_coding	0.606	7.53835E-44
AATF	RNF220	protein_coding	0.694	4.03465E-62
AATF	SAE1	protein_coding	0.683	1.83833E-59
AATF	RASSF1	protein_coding	0.62	2.36376E-46
AATF	EXOSC10	protein_coding	0.617	6.302E-46
AATF	SHKBP1	protein_coding	0.641	1.72257E-50
AATF	ZCCHC17	protein_coding	0.661	1.60824E-54
AATF	E2F4	protein_coding	0.608	3.45021E-44
AATF	TIMM50	protein_coding	0.619	2.99392E-46
AATF	HNRNPA2B1	protein_coding	0.628	7.21609E-48
AATF	MRPL53	protein_coding	0.664	2.92053E-55
AATF	TMEM106C	protein_coding	0.632	1.11951E-48
AATF	POC1A	protein_coding	0.615	2.12586E-45
AATF	TPX2	protein_coding	0.622	7.87609E-47
AATF	CCDC59	protein_coding	0.633	6.30455E-49
AATF	QARS	protein_coding	0.645	3.86395E-51
AATF	PYGO2	protein_coding	0.702	3.01942E-64

AATF	FBL	protein_coding	0.631	1.502E-48
AATF	MARS	protein_coding	0.614	2.66524E-45
AATF	METTL5	protein_coding	0.676	5.66396E-58
AATF	FAM104A	protein_coding	0.643	8.13756E-51
AATF	WDR77	protein_coding	0.606	6.18803E-44
AATF	ZBED8	protein_coding	0.602	3.49917E-43
AATF	CSE1L	protein_coding	0.694	4.13338E-62
AATF	HARS	protein_coding	0.603	2.52461E-43
AATF	DAZAP1	protein_coding	0.711	1.37109E-66
AATF	SSBP1	protein_coding	0.614	2.92254E-45
AATF	SRRT	protein_coding	0.646	1.96591E-51
AATF	TMEM14B	protein_coding	0.603	2.08722E-43
AATF	SNRPB2	protein_coding	0.668	3.40506E-56
AATF	HNRNPU	protein_coding	0.682	2.88461E-59
AATF	NUP85	protein_coding	0.733	1.57706E-72
AATF	DYNLL1	protein_coding	0.625	2.9456E-47
AATF	SLC52A2	protein_coding	0.627	1.01485E-47
AATF	CNIH4	protein_coding	0.617	7.81835E-46
AATF	PKMYT1	protein_coding	0.677	4.56291E-58
AATF	DHX30	protein_coding	0.652	1.19494E-52
AATF	USP36	protein_coding	0.602	3.82576E-43
AATF	RPL19	protein_coding	0.722	1.71979E-69
AATF	GPN1	protein_coding	0.667	5.92239E-56
AATF	CYTH2	protein_coding	0.601	5.75191E-43
AATF	ANAPC7	protein_coding	0.699	1.92227E-63
AATF	NFYC	protein_coding	0.603	2.49418E-43
AATF	PSMD3	protein_coding	0.783	5.87447E-89
AATF	CCNB1	protein_coding	0.685	5.08055E-60
AATF	PRKRIP1	protein_coding	0.621	1.34091E-46
AATF	MRM2	protein_coding	0.663	5.7184E-55
AATF	RBMX	protein_coding	0.675	8.57421E-58
AATF	NELFB	protein_coding	0.628	8.21553E-48
AATF	SFSWAP	protein_coding	0.634	3.79589E-49
AATF	KCMF1	protein_coding	0.615	1.98293E-45
AATF	SMARCAL1	protein_coding	0.666	1.09277E-55
AATF	SNRPA	protein_coding	0.694	3.05053E-62
AATF	RRP9	protein_coding	0.745	2.30274E-76
AATF	AP2B1	protein_coding	0.623	5.02699E-47
AATF	CKLF	protein_coding	0.605	9.32471E-44
AATF	JMJD4	protein_coding	0.602	3.26904E-43
AATF	ORMDL2	protein_coding	0.605	1.24177E-43
AATF	FAM50A	protein_coding	0.667	6.64105E-56
AATF	DHX16	protein_coding	0.638	9.75211E-50
AATF	RUFY1	protein_coding	0.643	8.29664E-51

AATF	C20orf27	protein_coding	0.653	5.73057E-53
AATF	XRCC6	protein_coding	0.714	3.01248E-67
AATF	PSMG3	protein_coding	0.634	5.36388E-49
AATF	MCRS1	protein_coding	0.724	5.73753E-70
AATF	BUD23	protein_coding	0.657	1.21514E-53
AATF	NUDT1	protein_coding	0.677	3.6219E-58
AATF	GPANK1	protein_coding	0.637	1.38271E-49
AATF	RPP30	protein_coding	0.627	9.79968E-48
AATF	NUP93	protein_coding	0.632	1.28795E-48
AATF	SLC25A39	protein_coding	0.68	6.77606E-59
AATF	DNTTIP1	protein_coding	0.715	1.4901E-67
AATF	C1orf35	protein_coding	0.71	3.44184E-66
AATF	CCT4	protein_coding	0.708	8.6195E-66
AATF	OXLD1	protein_coding	0.657	9.55531E-54
AATF	AAR2	protein_coding	0.621	1.62135E-46
AATF	HM13	protein_coding	0.607	4.25271E-44
AATF	DVL2	protein_coding	0.617	7.29841E-46
AATF	ATIC	protein_coding	0.705	5.72415E-65
AATF	PTGES3	protein_coding	0.704	8.19555E-65
AATF	GEMIN6	protein_coding	0.601	5.16342E-43
AATF	TRAIP	protein_coding	0.683	1.54429E-59
AATF	KTI12	protein_coding	0.664	3.91085E-55
AATF	PSMB4	protein_coding	0.682	2.30534E-59
AATF	GOSR2	protein_coding	0.645	3.41437E-51
AATF	NCBP2	protein_coding	0.605	9.56135E-44
AATF	METTL13	protein_coding	0.624	4.37442E-47
AATF	EIF1	protein_coding	0.669	2.66703E-56
AATF	EZH1	protein_coding	0.601	5.96125E-43
AATF	MIEN1	protein_coding	0.735	3.86036E-73
AATF	ZNF282	protein_coding	0.624	4.49959E-47
AATF	SNX17	protein_coding	0.652	1.28238E-52
AATF	NELFCD	protein_coding	0.623	6.49756E-47
AATF	ZNF581	protein_coding	0.615	1.6162E-45
AATF	POLR3F	protein_coding	0.619	3.35864E-46
AATF	PDCD5	protein_coding	0.634	4.44846E-49
AATF	TRIM65	protein_coding	0.681	3.66875E-59
AATF	GRB2	protein_coding	0.64	3.62231E-50
AATF	PTPN23	protein_coding	0.621	1.47288E-46
AATF	LSM8	protein_coding	0.614	3.08306E-45
AATF	RDM1	protein_coding	0.601	5.89569E-43
AATF	DCTN2	protein_coding	0.729	1.97124E-71
AATF	LAMTOR1	protein_coding	0.607	5.55114E-44
AATF	RIC8A	protein_coding	0.645	2.46578E-51
AATF	ILKAP	protein_coding	0.657	7.94845E-54

AATF	TADA2A	protein_coding	0.641	1.79694E-50
AATF	STIP1	protein_coding	0.712	1.0901E-66
AATF	SAP30BP	protein_coding	0.765	9.01432E-83
AATF	AMZ2	protein_coding	0.685	4.07243E-60
AATF	MED25	protein_coding	0.617	7.22425E-46
AATF	UBE2Z	protein_coding	0.719	1.26054E-68
AATF	PIGC	protein_coding	0.66	1.98213E-54
AATF	MELK	protein_coding	0.636	2.02628E-49
AATF	GLRX3	protein_coding	0.609	1.88661E-44
AATF	ADRM1	protein_coding	0.642	1.08829E-50
AATF	XRCC1	protein_coding	0.648	7.56143E-52
AATF	CENPH	protein_coding	0.672	6.4091E-57
AATF	C8orf33	protein_coding	0.603	2.19074E-43
AATF	ARFIP2	protein_coding	0.615	1.65414E-45
AATF	PNO1	protein_coding	0.606	6.7535E-44
AATF	PLK1	protein_coding	0.64	3.61299E-50
AATF	CFAP36	protein_coding	0.604	1.50279E-43
AATF	EFTUD2	protein_coding	0.827	1.8123E-107
AATF	POLR2H	protein_coding	0.632	9.75306E-49
AATF	ARL16	protein_coding	0.662	1.00754E-54
AATF	TRMT1	protein_coding	0.684	8.09052E-60
AATF	VPS16	protein_coding	0.657	1.21296E-53
AATF	SYMPK	protein_coding	0.634	4.23607E-49
AATF	IMPDH2	protein_coding	0.64	2.87857E-50
AATF	SPATS2	protein_coding	0.66	2.04044E-54
AATF	BCL2L12	protein_coding	0.61	1.226E-44
